# Mechanical stability and thermodynamic properties of GeP and $\hbox {GeP}_{3}$ as battery anode materials from first principles

**DOI:** 10.1038/s41598-026-36336-1

**Published:** 2026-01-23

**Authors:** Duc Toan Truong, Nguyen-Hieu Hoang, Chi M. Phan, An-Giang Nguyen, Thuat T. Trinh

**Affiliations:** 1 Laboratory for Chemical Computation and Modeling, Institute for Computational Science and Artificial Intelligence, Van Lang University, Ho Chi Minh City, 70000 Vietnam; 2 Faculty of Applied Technology, Van Lang School of Technology, Van Lang University, Ho Chi Minh City, 70000 Vietnam; 3 Department of Materials and Nanotechnology, SINTEF Industry, Trondheim, NO-7034 Norway; 4 Discipline of Chemical Engineering, WASM, Curtin University, Perth, WA 6045 Australia; 5 College of Engineering and Computer Science, VinUniversity, Hanoi, 10000 Vietnam; 6 Center for Environmental Intelligence, VinUniversity, Hanoi, 10000 Vietnam; 7 Porelab, Department of Chemistry and Biomedical Science, Norwegian University of Science and Technology, NTNU, Trondheim, NO-7491 Norway

**Keywords:** Engineering, Materials science

## Abstract

The demand for high-capacity anode materials beyond conventional graphite has intensified research into alternative candidates for next-generation lithium-ion and sodium-ion batteries. Germanium phosphides emerge as promising materials, combining germanium’s high theoretical capacity with phosphorus’s structural versatility and potential for improved cycling stability. We employ first-principles density functional theory calculations to systematically investigate the mechanical, electronic, and thermodynamic properties of three GeP polymorphs (monoclinic, tetragonal, cubic) and rhombohedral $\hbox {GeP}_{3}$ as potential anode materials. Our comprehensive analysis reveals that polymorphism critically influences anode performance through distinct mechanical and electronic characteristics. GeP-cubic exhibits mechanical instability, rendering it unsuitable for practical applications. GeP-tetragonal shows the highest stiffness (bulk modulus 79.4 GPa, Young’s modulus 170.7 GPa) but pronounced brittleness (Pugh’s ratio K/G = 1.06), potentially limiting cycling durability. GeP-monoclinic offers greater mechanical compliance (bulk modulus 32.1 GPa) but suffers from extreme elastic anisotropy (universal anisotropy index A$_U$ = 7.90), which may lead to non-uniform stress distribution and structural degradation during cycling. In contrast, $\hbox {GeP}_{3}$ demonstrates an optimal balance of properties with intermediate mechanical stiffness (bulk modulus 61.0 GPa, Young’s modulus 121.6 GPa), low elastic anisotropy (A$_U$ = 0.77). Electronic structure calculations reveal metallic conductivity for GeP-tetragonal, GeP-cubic, and $\hbox {GeP}_{3}$, ensuring efficient charge transport during battery operation. These findings establish $\hbox {GeP}_{3}$ as the most promising candidate among the studied materials, offering balanced mechanical resilience, thermal robustness, and isotropic properties essential for stable long-term cycling performance in practical battery applications.

## Introduction

The rapid advancement of portable electronic devices and electric vehicles has intensified the demand for high-performance energy storage systems with superior capacity, stability, and safety characteristics^1^. Lithium-ion batteries (LIBs) have dominated the rechargeable battery market for decades, yet their theoretical capacity limitations and resource scarcity concerns have motivated extensive research into alternative anode materials beyond conventional graphite^2^. Recent developments in solid-state battery technologies have further emphasized the need for advanced electrode materials that can operate effectively with novel electrolyte systems, including composite solid electrolytes with enhanced ionic conductivity and mechanical stability^3,4^. Among the promising candidates, Group IV-V layered materials have emerged as particularly attractive due to their unique structural properties and high theoretical capacities for lithium and sodium storage^5^.

Germanium phosphide compounds, particularly GeP and $\hbox {GeP}_{3}$, represent a fascinating class of anode materials that combine the advantageous properties of both germanium and phosphorus elements. Germanium, with its high theoretical capacity of 1624 mAh g$^{-1}$ and favorable lithium alloying behavior, has been extensively studied as a high-capacity anode material^2^. Phosphorus, on the other hand, offers an exceptional theoretical capacity of 2596 mAh g$^{-1}$ for sodium-ion batteries through conversion reactions^6^. The combination of these elements in germanium phosphide compounds potentially offers synergistic effects that could overcome individual limitations while maintaining high storage capacities. Recent advances in germanium-phosphorus based materials have demonstrated their potential for alternative battery chemistries, including potassium-ion batteries where $\hbox {GeP}_{5}$ materials show promising electrochemical performance with enhanced cycling stability through appropriate carbon coating strategies^7,8^.

Recent experimental investigations have demonstrated the promising electrochemical performance of germanium phosphide materials. Nam et al.^9^ synthesized layered $\hbox {GeP}_{3}$ using a solid-state method and reported remarkable reversible capacities of 1526 mAh g$^{-1}$ for LIBs and 984 mAh g$^{-1}$ for sodium-ion batteries (SIBs), with high initial Coulombic efficiencies exceeding 80%. The material exhibited complex multi-step reaction mechanisms involving topotactic transitions, conversion reactions, and alloying processes during lithiation/delithiation cycles. Similarly, Tseng et al.^10^ developed mesoporous germanium phosphide microspheres that delivered exceptional rate capabilities, achieving 370 mAh g$^{-1}$ at an ultra-high current density of 72 A g$^{-1}$.

The structural diversity of germanium phosphide compounds offers unique opportunities for tailoring electrochemical properties. Layered GeP has been shown to exhibit anisotropic lithium diffusion properties, with theoretical calculations predicting diffusion rates approximately 1000 times faster than graphene^5,11^. However, practical implementation faces challenges related to large volume changes during cycling, which can lead to electrode pulverization and capacity degradation^12^. Strategies such as voltage window optimization and nanostructuring have been employed to mitigate these issues and improve cycling stability. Additionally, the development of advanced cathode materials through doping strategies, such as aluminum and boron co-doping in nickel-rich materials, has shown significant improvements in electrochemical stability and cycling performance^13^, highlighting the importance of comprehensive material optimization approaches.

From a theoretical perspective, density functional theory (DFT) calculations have proven invaluable for understanding the fundamental properties of battery materials and predicting their electrochemical behavior^14,15^. Recent advances in computational materials science have demonstrated the reliability of DFT methods for predicting structural, mechanical, and thermodynamic properties across diverse material systems, including complex crystalline phases^16,17^. First-principles molecular dynamics simulations have further enhanced our understanding of atomic-scale processes and reaction mechanisms in materials containing germanium and related Group IV elements^18,19^. DFT studies on germanium phosphide systems have primarily focused on 2D monolayer structures, revealing insights into electronic structures, lithium diffusion pathways, and phase stability for these low-dimensional systems^5^. However, comprehensive computational investigations covering the full spectrum of structural, mechanical, electronic, and thermodynamic properties of bulk (3D) crystalline GeP and $\hbox {GeP}_{3}$ phases remain limited in the literature, particularly regarding mechanical properties that are critical for practical battery anode applications where bulk material behavior determines cycling stability.

The mechanical properties of anode materials play a crucial role in determining their cycling stability and practical viability. Elastic constants and mechanical moduli provide fundamental insights into material deformation behavior under stress, which is particularly relevant for materials experiencing significant volume changes during lithiation/delithiation processes^20^. Understanding these mechanical characteristics through first-principles calculations can guide the design of more robust electrode architectures and predict long-term cycling performance.

Thermodynamic stability assessment is equally important for predicting phase behavior under operating conditions and identifying potential decomposition pathways. The importance of temperature-dependent electrochemical behavior has been further emphasized by recent developments in electrolyte engineering for sub-zero temperature battery operation, where understanding material stability across wide temperature ranges becomes critical for practical implementation^21^. Advanced computational approaches, including reactive force field methods and small system thermodynamics, have proven effective for calculating thermodynamic properties of complex reactions and phase transitions under extreme conditions^22^.

Electronic structure analysis through band structure and density of states calculations provides essential information about charge transport properties, which directly influence rate capabilities and electrochemical kinetics^23^. The semiconducting nature of germanium phosphide compounds and their electronic band gaps significantly impact their conductivity and may require optimization strategies such as doping or composite formation with conductive matrices^24^.

Despite the promising experimental results and growing interest in germanium phosphide anode materials, a comprehensive theoretical framework that systematically compares the fundamental properties of different germanium phosphide phases is still lacking. Such a framework would provide valuable insights for rational material design and optimization strategies. Furthermore, the relationship between structural characteristics and electrochemical performance requires deeper understanding through detailed computational analysis. Notably, while polymorphism in GeP is known to exist, no systematic computational study has comprehensively compared all three polymorphs (monoclinic, tetragonal, and cubic) alongside $\hbox {GeP}_{3}$ within a unified framework to understand how crystal structure fundamentally governs material properties despite identical composition.

In this work, we present a comprehensive first-principles study of bulk GeP polymorphs and $\hbox {GeP}_{3}$ crystals as potential anode materials for lithium-ion batteries. Using DFT calculations, we systematically investigate their structural, mechanical, electronic, and thermodynamic properties to establish a fundamental understanding of their behavior and potential for battery applications. Our study addresses the critical gap in bulk mechanical property data by providing comprehensive analysis of elastic constants, moduli, and anisotropy indices for all phases. We establish a quantitative framework that connects structural characteristics, mechanical properties, electronic structure, and thermodynamic behavior to battery anode performance, revealing how polymorphism fundamentally governs material properties and identifying mechanical stability as the primary determinant for practical applications. Through systematic comparison, we identify mechanically unstable phases and quantify the capacity-versus-stability trade-off, enabling application-specific material selection. Our study aims to bridge the gap between experimental observations and theoretical predictions, providing quantitative design guidelines that can guide future experimental efforts and material optimization strategies. The results contribute to the growing body of knowledge on advanced anode materials and support the development of next-generation energy storage systems with enhanced performance characteristics.

## Computational methods

The investigation focused on three experimentally reported GeP polymorphs and one $\hbox {GeP}_{3}$ phase. These include monoclinic GeP-mono (space group C2/m), tetragonal GeP-tetra (I4mm), and cubic GeP-cubic (F-43m), along with rhombohedral $\hbox {GeP}_{3}$ (R-3m). Initial atomic coordinates and lattice parameters were obtained from crystallographic databases and experimental literature^25,26^, providing realistic starting points for structural optimization.

Density functional theory (DFT) calculations were performed using the Vienna Ab initio Simulation Package (VASP)^27,28^ with the Perdew-Burke-Ernzerhof (PBE) generalized gradient approximation functional^29^. To accurately describe van der Waals interactions important for layered materials, DFT-D2 corrections^30^ were applied throughout all calculations. The projector-augmented wave method^31^ was employed with a plane-wave energy cutoff of 600 eV, which was determined through systematic convergence tests to ensure energy convergence within $\le$ 0.1 meV/atom and stress convergence within $\le$ 0.1 GPa (see Supporting Information, Section S1).

Brillouin zone sampling utilized $\Gamma$-centered Monkhorst-Pack k-point meshes. For structural optimizations, specific meshes were selected for each phase based on systematic convergence tests to ensure accurate reproduction of experimental lattice parameters: GeP-mono (monoclinic): 2$\times$9$\times$4; GeP-tetra (tetragonal): 9$\times$9$\times$6; GeP-cubic (cubic): 6$\times$6$\times$6; and $\hbox {GeP}_{3}$ (rhombohedral): 4$\times$4$\times$2. These meshes correspond to k-point spacing of approximately 0.030–0.043 (2$\pi$/a) and were verified to ensure convergence of both total energy (within 3.5 meV/atom relative to denser meshes) and stress tensors (differences < 1.3 GPa). The accuracy of these meshes is validated by the excellent agreement with experimental lattice parameters (deviations < 1.5%, as shown in Table 1). For electronic structure calculations (density of states and band structures), significantly denser k-point grids were employed to ensure proper convergence of electronic density of states features. Detailed convergence tests for $\hbox {GeP}_{3}$ are provided in the Supporting Information (Section S1), demonstrating that the selected meshes for geometry optimization provide a good balance between accuracy and computational efficiency while being sufficient to match experimental structural data.

The choice of PBE-D2 was validated through systematic comparison with other methods (PBE, PBE-D3, optB88-vdW) for all four phases studied, as detailed in the Supporting Information (Section S2, Tables S1 and S2). Across all phases, PBE-D2 consistently ranks among the best methods for reproducing experimental lattice parameters. For GeP-mono, PBE-D2 shows the best agreement (0.26% volume deviation), significantly outperforming other methods. For GeP-tetra and GeP-cubic, PBE-D2 also provides excellent or best agreement. For $\hbox {GeP}_{3}$, PBE-D2 provides excellent agreement with experimental lattice parameters, particularly for the in-plane parameter (0.36% deviation), which is critical for layered materials, and ranks among the best methods overall. PBE-D2 has been widely validated for Group IV-V layered materials, and its consistent performance across all phases makes it an appropriate choice for this comprehensive study. The PBE functional and its dispersion-corrected variants (PBE-D2, PBE-D3) have been extensively used by multiple research groups for GeP and $\hbox {GeP}_{3}$ systems^5,11,32–35^, demonstrating its reliability for both metallic and semiconducting germanium phosphide phases. The choice of PBE is appropriate for the mechanical and thermodynamic property calculations that are the primary focus of this work.

Structural relaxations simultaneously optimized atomic positions and lattice parameters with the convergence criteria of 10$^{-6}$ eV for electronic self-consistency and ionic forces below 0.01 eV/Å. The k-point meshes used for geometry optimizations were verified to be sufficient to reproduce experimental lattice parameters with excellent agreement (see Table 1). Following structural optimization, electronic properties were analyzed through density of states (DOS) and band structure calculations using enhanced precision settings. For accurate DOS calculations, significantly denser k-point grids were employed compared to structural optimizations, with k-point densities increased to ensure proper convergence of electronic density of states features. Projected DOS on atomic orbitals were computed to elucidate bonding characteristics and identify electronic contributions from germanium and phosphorus atoms.

To validate the electronic structure predictions and address the well-known band gap underestimation of standard GGA functionals, additional calculations were performed using the Tran-Blaha modified Becke-Johnson (TB-mBJ) meta-GGA functional^36^ for GeP-mono. The TB-mBJ functional provides significantly improved band gap predictions at computational cost substantially lower than hybrid functionals, making it particularly suitable for validating electronic properties. This approach has been demonstrated to provide band gap accuracy comparable to hybrid functionals such as HSE06 for semiconducting materials while maintaining computational efficiency.

In order to evaluate the elastic properties of the material, we used an established method outlined briefly here. The generalised Hooke’s Law for a linear elastic material is written as:

1 $$\begin{aligned} \left[ \begin{array}{l} \sigma _{1}=\sigma _{x x} \\ \sigma _{2}=\sigma _{y y} \\ \sigma _{3}=\sigma _{z z} \\ \sigma _{4}=\sigma _{y z} \\ \sigma _{5}=\sigma _{x z} \\ \sigma _{6}=\sigma _{x y} \end{array}\right] =\left[ \begin{array}{llllll} C_{11} & C_{12} & C_{13} & C_{14} & C_{15} & C_{16} \\ C_{21} & C_{22} & C_{23} & C_{24} & C_{25} & C_{26} \\ C_{31} & C_{32} & C_{33} & C_{34} & C_{35} & C_{36} \\ C_{41} & C_{42} & C_{43} & C_{44} & C_{45} & C_{46} \\ C_{51} & C_{52} & C_{53} & C_{54} & C_{55} & C_{56} \\ C_{61} & C_{62} & C_{63} & C_{64} & C_{65} & C_{66} \end{array}\right] \left[ \begin{array}{l} \varepsilon _{1}=\varepsilon _{x x} \\ \varepsilon _{2}=\varepsilon _{y y} \\ \varepsilon _{3}=\varepsilon _{z z} \\ \varepsilon _{4}=\varepsilon _{y z} \\ \varepsilon _{5}=\varepsilon _{x z} \\ \varepsilon _{6}=\varepsilon _{x y} \end{array}\right] \end{aligned}$$

This notation with only one subscript for the stress and strain, numbered from 1...6, is helpful as it allows the equations of anisotropic elasticity to be written in matrix form. The 36’s $C_{ij}$ are called the stiffnesses. The matrix of stiffnesses is called the stiffness matrix. This matrix can be inverted so that the strains are given explicitly in terms of the stresses:

2 $$\begin{aligned} \left[ \begin{array}{c} \varepsilon _{1} \\ \varepsilon _{2} \\ \varepsilon _{3} \\ \varepsilon _{4} \\ \varepsilon _{5} \\ \varepsilon _{6} \end{array}\right] =\left[ \begin{array}{cccccc} S_{11} & S_{12} & S_{13} & S_{14} & S_{15} & S_{16} \\ & S_{22} & S_{23} & S_{24} & S_{25} & S_{26} \\ & & S_{33} & S_{34} & S_{35} & S_{36} \\ & & & S_{44} & S_{45} & S_{46} \\ & & & & S_{55} & S_{56} \\ & & & & & S_{66} \end{array}\right] \left[ \begin{array}{c} \sigma _{1} \\ \sigma _{2} \\ \sigma _{3} \\ \sigma _{4} \\ \sigma _{5} \\ \sigma _{6} \end{array}\right] \end{aligned}$$

The stiffness constants $C_{ij}$ and constituents of the compliance tensor $S_{ij}$ were calculated using VASP. From the calculated $C_{ij}$ and $S_{ij}$, the polycrystalline corresponding bulk modulus *K* and shear modulus *G* are determined using the Voigt-Reuss-Hill (VRH) approximation^37^, as shown in Eqs. 3 and 4. In these equations, the underscripts *R* and *V* denote Reuss and Voigt bounds, respectively.

3 $$\begin{aligned} \begin{aligned}&K_{R}= & \frac{1}{S_{11}+S_{22}+S_{33}+2(S_{12}+S_{23}+S_{31})} \\&K_{V}= & \frac{C_{11}+C_{22}+C_{33}+2(C_{12}+C_{23}+C_{31})}{9} \\&K= & \frac{K_{R}+K_{V}}{2} \end{aligned} \end{aligned}$$

4 $$\begin{aligned} \begin{aligned}&G_{R}= & \frac{15}{4(S_{11}+S_{22}+S_{33})-4(S_{12}+S_{23}+S_{31})+3(S_{44}+S_{55}+S_{66})} \\&G_{V}= & \frac{C_{11}+C_{22}+C_{33}-(C_{12}+C_{23}+C_{31})+3(C_{44}+C_{55}+C_{66})}{15} \\&G= & \frac{G_{R}+G_{V}}{2} \end{aligned} \end{aligned}$$

The Young’s modulus, *E*, and Poisson’s ratio, $\nu$, for an isotropic material can then be estimated by:

5 $$\begin{aligned} E = \frac{9KG}{3K+G}, \; \nu = \frac{3K-2G}{6K+2G} \end{aligned}$$

Thermodynamic properties were evaluated using the Debye model approach based on calculated elastic constants to determine Debye temperatures and estimate thermal properties^38^. This approach offers reliable estimates of key thermodynamic parameters and enables comparative analysis between different polymorphs within a consistent theoretical framework^16,17^. Dynamic stability was rigorously confirmed through comprehensive phonon dispersion calculations using the finite displacement method as implemented in Phonopy^39^, performed on optimized supercells (72–96 atoms) with high-precision VASP force calculations (ENCUT = 600 eV, EDIFF = 1$\times$10$^{-8}$ eV); complete phonon band structures and phonon density of states confirming the absence of imaginary frequencies are presented in the Supporting Information (Section S4).

## Results and discussion

This section presents a comprehensive analysis of the structural, mechanical, electronic, and thermodynamic properties of GeP and $\hbox {GeP}_{3}$ obtained from first-principles DFT calculations. The results provide fundamental insights into the behavior of these materials and their potential applications as anode materials in lithium-ion batteries.

### Structural properties

**Fig. 1:**
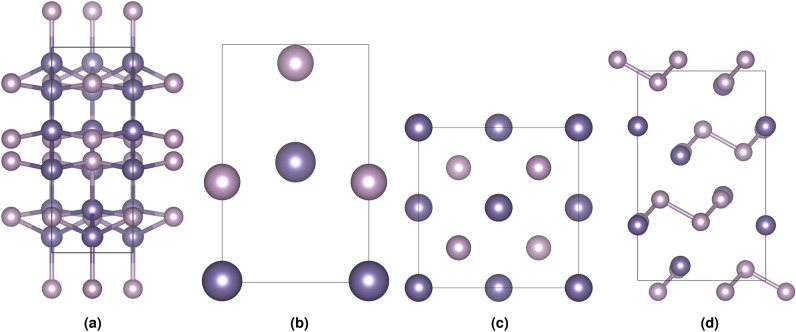
Crystal structures of GeP polymorphs and $\hbox {GeP}_{3}$. The structures show ball-and-stick representations with unit cells indicated. Ge atoms are shown in darker color and P atoms in lighter color.

The optimized crystal structures of GeP and $\hbox {GeP}_{3}$ obtained from DFT calculations reveal distinct crystallographic characteristics reflecting their different stoichiometries and bonding environments (Fig. 1). For clarity, we refer to the three GeP polymorphs as GeP-mono (C2/m), GeP-tetra (I4mm), and GeP-cubic (F-43m) throughout this analysis. To validate our computational approach, we systematically compared the optimized structures against experimental data using crystallographic databases and literature sources.

Among the GeP polymorphs, the monoclinic GeP-mono represents the most stable configuration with lattice parameters a = 15.161 Å, b = 3.652 Å, c = 9.153 Å, and lattice angle $\beta$ = 100.733${\circ }$. This layered structure contains 24 atoms per unit cell arranged in a characteristic Group IV-V bonding pattern, with Ge-P bond lengths ranging from 2.35 to 2.48 Å. The relatively large interlayer spacing along the b-axis suggests favorable pathways for lithium intercalation, while the germanium atoms adopt primarily tetrahedral coordination with phosphorus neighbors.

The tetragonal GeP-tetra polymorph exhibits a more compact structure with identical in-plane lattice parameters (a = b = 3.526 Å) and c = 5.667 Å, reflecting the higher symmetry constraints. This configuration results in a denser packing arrangement that significantly influences the mechanical properties discussed in subsequent sections. In contrast, the cubic GeP-cubic variant presents a high-symmetry structure, though as will be demonstrated through mechanical analysis, this polymorph suffers from inherent stability issues.

**Table 1:** Comparison between theoretical calculated crystal parameters and experimental values for GeP and GeP3 phases. Experimental data are provided for comparison, with values in parentheses indicating the relative deviation from the experimental data. Angles are omitted due to negligible differences relative to experiment. Experimental values for GeP are taken from ref^25^ and for $\hbox {GeP}_{3}$ from ref^26^.

Phase	Space group	a (Å)		b (Å)		c (Å)		V (Å$^3$)	
		Exp	Calc (dev%)	Exp	Calc (dev%)	Exp	Calc (dev%)	Exp	Calc (dev%)
GeP-mono	C2/m (12)	15.140	15.161 (0.14)	3.638	3.652 (0.40)	9.190	9.153 (0.40)	496.7	498.0 (0.26)
GeP-tetra	I4mm (107)	3.544	3.526 (0.51)	3.544	3.526 (0.51)	5.581	5.667 (1.53)	70.1	70.5 (0.52)
GeP-cubic	F-43m (216)	5.463	5.575 (2.03)	5.463	5.575 (2.03)	5.463	5.575 (2.03)	163.0	173.3 (6.09)
GeP3	R-3m (166)	7.050	7.075 (0.36)	7.050	7.075 (0.36)	9.932	9.633 (3.05)	427.5	417.7 (2.33)

Moving to the phosphorus-rich composition, $\hbox {GeP}_{3}$ adopts a rhombohedral structure with lattice parameters a = b = 7.075 Å and c = 9.633 Å. The hexagonal symmetry in the ab-plane combined with the distinct c-axis creates a layered architecture fundamentally different from the GeP polymorphs. The unit cell accommodates 24 atoms with a 1:3 Ge:P stoichiometric ratio, resulting in shorter average Ge-P bond lengths (2.32–2.45.32.45 Å) that indicate stronger covalent interactions due to the increased phosphorus content. This bonding environment establishes germanium atoms in predominantly tetrahedral coordination while phosphorus atoms exhibit varied coordination numbers from two to four, creating a robust three-dimensional covalent network.

The structural validation presented in Table 1 demonstrates excellent agreement between our calculated parameters and experimental values. The deviations remain remarkably small across all phases, with GeP-mono showing lattice parameter deviations below 0.4% and a volume deviation of only 0.26%, GeP-tetra within 1.5% for lattice parameters and 0.52% for volume, and GeP3 within 3.1% for lattice parameters and 2.33% for volume. The GeP-cubic polymorph shows slightly larger deviations of 2.03% for lattice parameters and 6.09% for volume, which may reflect the inherent metastability of this high-symmetry phase. Notably, the volume comparisons provide an integrated measure of structural accuracy that accounts for the combined effects of all lattice parameter variations, and the excellent agreement (most volume deviations <3%) confirms the reliability of our computational methodology and provides confidence in the subsequent property predictions. The excellent agreement between experimental and calculated volumes validates our structural models for predicting volumetric expansion during lithiation. The $\hbox {GeP}_{3}$ phase, with its intermediate volume and higher phosphorus content, provides a higher density of electroactive sites that could participate in conversion reactions, potentially delivering superior theoretical capacities. The layered nature of both compounds provides natural accommodation for volume changes during electrochemical cycling, though the specific anisotropic expansion behaviors will depend on the directional mechanical properties analyzed in the following section.

### Mechanical properties

The mechanical properties of GeP and $\hbox {GeP}_{3}$ were systematically investigated through comprehensive elastic constant calculations using the stress-strain method. Understanding the mechanical response is crucial for battery applications where materials experience significant volume changes during lithiation/delithiation cycles, making mechanical stability and deformation behavior key factors determining cycling performance and electrode integrity. Our analysis encompasses all three GeP polymorphs (GeP-mono, GeP-tetra, GeP-cubic) and $\hbox {GeP}_{3}$, revealing striking differences in mechanical behavior that directly influence their practical viability as anode materials.

The calculated elastic constant matrices presented in Table 2 reveal important differences in mechanical behavior among the studied structures, with implications ranging from exceptional stiffness to complete mechanical instability. GeP-mono demonstrates significant anisotropy characteristic of layered monoclinic materials, with C$_{22}$ (165.2 GPa) substantially exceeding C$_{11}$ (41.9 GPa) and C$_{33}$ (110.7 GPa). The substantial off-diagonal terms, particularly C$_{16}$ (22.5 GPa) and C$_{36}$ (23.2 GPa), indicate strong coupling between normal and shear deformations that reflects the directional bonding network. Despite this anisotropy, the relatively moderate elastic constants suggest sufficient structural compliance to accommodate volume changes during lithiation without excessive stress buildup.

GeP-tetra emerges as the mechanically superior polymorph, exhibiting exceptionally high elastic constants with C$_{11}$ = C$_{22}$ = 245.6 GPa and C$_{33}$ = 161.7 GPa, establishing it as the stiffest structure among all investigated materials. The perfect in-plane symmetry reflects the tetragonal crystal symmetry, while the remarkably high shear modulus C$_{44}$ = 120.9 GPa demonstrates exceptional resistance to shear deformation. The absence of off-diagonal coupling terms confirms the high degree of structural symmetry, though the contrast with lower C$_{55}$ = C$_{66}$ = 43.5 GPa values reveals pronounced anisotropic mechanical behavior with exceptional in-plane rigidity but reduced resistance to specific shear modes.

Conversely, GeP-cubic exhibits mechanical instability that eliminates its practical viability despite the appealing high-symmetry cubic structure. The large negative shear elastic constants (C$_{44}$ = −150.6 GPa, C$_{55}$ = −151.1 GPa, C$_{66}$ = −151.0 GPa) fundamentally violate stability criteria, while the extremely low diagonal terms (C$_{11}$ = 4.3 GPa, C$_{22}$ = 1.7 GPa, C$_{33}$ = 1.7 GPa) indicate negligible resistance to normal stresses. This instability likely originates from geometric frustration inherent in forcing Ge-P covalent bonding into a cubic framework, resulting in a structure that cannot sustain mechanical stress.

**Table 2:** Calculated elastic constants $C_{ij}$ (GPa) for GeP polymorphs and $\hbox {GeP}_{3}$. GeP-cubic exhibits mechanical instability.

GeP-mono						
$C_{ij}$	1	2	3	4	5	6
1	41.9	37.8	11.9	−1.4	−0.2	22.5
2	37.8	165.2	16.4	−1.6	−0.0	14.3
3	11.9	16.4	110.7	−1.5	−0.1	23.2
4	−1.4	−1.6	−1.5	43.3	16.4	0.1
5	−0.2	−0.0	−0.1	16.4	28.5	−0.1
6	22.5	14.3	23.2	0.1	−0.1	25.1
GeP-tetra						
$C_{ij}$	1	2	3	4	5	6
1	245.6	27.1	6.7	0.0	0.0	0.0
2	27.1	245.6	6.7	0.0	0.0	0.0
3	6.7	6.7	161.7	0.0	0.0	0.0
4	0.0	0.0	0.0	120.9	0.0	0.0
5	0.0	0.0	0.0	0.0	43.5	0.0
6	0.0	0.0	0.0	0.0	0.0	43.5
GeP-cubic – Mechanically Unstable						
$C_{ij}$	1	2	3	4	5	6
1	4.3	65.3	65.4	−0.3	0.2	0.0
2	65.3	1.7	64.3	−0.3	0.1	0.0
3	65.4	64.3	1.7	−0.3	0.1	0.0
4	−0.3	−0.3	−0.3	−150.6	−0.4	−0.2
5	0.2	0.1	0.1	−0.4	−151.1	0.0
6	0.0	0.0	0.0	−0.2	0.0	−151.0
GeP3						
$C_{ij}$	1	2	3	4	5	6
1	175.7	43.2	19.8	0.2	12.2	0.1
2	43.2	177.1	21.4	0.2	−11.8	0.1
3	19.8	21.4	77.8	0.2	0.1	0.1
4	0.2	0.2	0.2	66.5	−0.2	11.9
5	12.2	−11.8	0.1	−0.2	46.3	−0.2
6	0.1	0.1	0.1	11.9	−0.2	46.2

The phosphorus-rich $\hbox {GeP}_{3}$ presents a distinctly different anisotropy pattern that reflects its rhombohedral symmetry, with nearly identical in-plane constants C$_{11}$ (175.7 GPa) and C$_{22}$ (177.1 GPa) substantially exceeding the out-of-plane value C$_{33}$ (77.8 GPa). This intermediate mechanical stiffness positions $\hbox {GeP}_{3}$ between the compliant GeP-mono and exceptionally stiff GeP-tetra, while the reduced off-diagonal coupling terms indicate less mechanical coupling between deformation modes.

This comparative analysis establishes a clear mechanical hierarchy among the stable phases, with GeP-tetra demonstrating the highest stiffness, $\hbox {GeP}_{3}$ exhibiting intermediate properties, and GeP-mono representing the most compliant structure. From a battery application perspective, this hierarchy presents important trade-offs between mechanical integrity and accommodation of lithiation-induced volume changes. While GeP-tetra’s exceptional stiffness might better resist structural degradation, it could generate excessive stress during cycling that leads to particle fracture. Conversely, GeP-mono’s compliance may facilitate volume accommodation but potentially compromise long-term structural integrity. The intermediate properties of $\hbox {GeP}_{3}$, combined with more isotropic behavior, suggest it may provide an optimal balance between mechanical stability and cycling accommodation.

The mechanical moduli derived through Voigt-Reuss-Hill averaging, summarized in Table 3, reveal the practical implications of the elastic constant variations and establish clear performance differentiation among the stable phases. GeP-tetra demonstrates exceptional mechanical performance with a bulk modulus of 79.4 GPa, shear modulus of 74.8 GPa, and Young’s modulus of 170.7 GPa, representing approximately threefold increases over GeP-mono across all parameters. The low Pugh’s ratio (K/G = 1.06) indicates exceptionally brittle behavior with strong resistance to both volumetric and shear deformation, suggesting limited accommodation of volume changes during cycling despite its high strength.

Occupying the intermediate mechanical regime, $\hbox {GeP}_{3}$ exhibits a bulk modulus of 61.0 GPa and shear modulus of 52.1 GPa that systematically fall between GeP-mono’s lower values (32.1 and 26.8 GPa, respectively) and GeP-tetra’s exceptional stiffness. The resulting Young’s modulus of 121.6 GPa represents approximately double that of GeP-mono, establishing $\hbox {GeP}_{3}$ as substantially stiffer while remaining more deformable than GeP-tetra. This intermediate positioning suggests $\hbox {GeP}_{3}$ may offer an optimal compromise between structural integrity and volume accommodation during electrochemical cycling.

The most compliant behavior observed in GeP-mono, while potentially appearing disadvantageous from a pure mechanical strength perspective, may actually provide benefits for battery applications where volume changes during lithiation can generate substantial internal stresses. The lower moduli could facilitate stress relaxation and volume accommodation, potentially extending cycle life by reducing particle fracture and maintaining electrical connectivity. However, this enhanced compliance must be balanced against concerns regarding structural degradation over extended cycling periods.

**Table 3:** Calculated mechanical moduli for GeP polymorphs and $\hbox {GeP}_{3}$: Bulk modulus *K*, Shear modulus *G*, Young’s modulus *E*, Poisson’s ratio $\nu$, and Pugh’s ratio *K*/*G*. Literature values from previous DFT calculations are included for comparison. GeP-cubic is mechanically unstable based on its elastic constants; moduli are therefore not reported.

Phase	Source	K (GPa)	G (GPa)	E (GPa)	$\nu$	K/G
Bulk phases (3D)						
GeP-mono	This work	32.1	26.8	62.9	0.173	1.20
GeP-tetra	This work	79.4	74.8	170.7	0.142	1.06
GeP-cubic	This work	–	–	–	–	–
$\hbox {GeP}_{3}$	This work	61.0	52.1	121.6	0.168	1.17
$\hbox {GeP}_{3}$	Ref^33^.	77.3$^a$	–	–	–	–
Phase	Source	E (GPa)$^b$ $\nu$				
Monolayer phases (2D) – for reference only						
GeP ($\alpha$)	Ref^35^.	25.6 0.23				
GeP ($\beta$)	Ref^35^.	35.4 0.20				
$\hbox {GeP}_{3}$	Ref^40^.	44.4 (N/m)$^c$				
$^a$B$_{VRH}$ (Voigt-Reuss-Hill bulk modulus for polycrystal)						
$^b$In-plane Young’s modulus E$_{xx}$ or E$_{yy}$ for 2D monolayer (GPa)						
$^c$2D in-plane Young’s modulus E$_y$ for monolayer (N/m)						

Comparison with available literature data provides validation of our mechanical property calculations. For bulk rhombohedral $\hbox {GeP}_{3}$, our calculated bulk modulus (K = 61.0 GPa) is lower than the value reported by Kim et al.^33^ (B$_{VRH}$ = 77.3 GPa). This difference can be attributed to variations in the crystalline structure used in the simulations. Kim et al. employed a different structural model or relaxation procedure that may have resulted in a more compact structure with higher stiffness. Such variations are common in DFT studies of layered materials where interlayer spacing and structural relaxation can significantly influence mechanical properties. Nevertheless, both values fall within the expected range for Group IV-V phosphide compounds, and the qualitative mechanical behavior remains consistent. The monolayer values from literature^35,40^ are provided for reference, showing that 2D systems exhibit significantly reduced stiffness compared to their bulk counterparts, as expected from the loss of interlayer bonding.

The Poisson’s ratio values provide additional mechanistic insights into deformation behavior, with GeP-tetra exhibiting the lowest value ($\nu$ = 0.142) that reflects its strong resistance to lateral strain under uniaxial stress. This behavior aligns with the exceptional stiffness demonstrated in other mechanical parameters, while $\hbox {GeP}_{3}$ shows intermediate behavior ($\nu$ = 0.168) and GeP-mono displays the highest Poisson’s ratio ($\nu$ = 0.173), indicating greater propensity for lateral deformation under axial stress. All values remain well below the 0.5 threshold characteristic of incompressible materials, confirming the covalently bonded nature of these germanium phosphides.

**Fig. 2:**
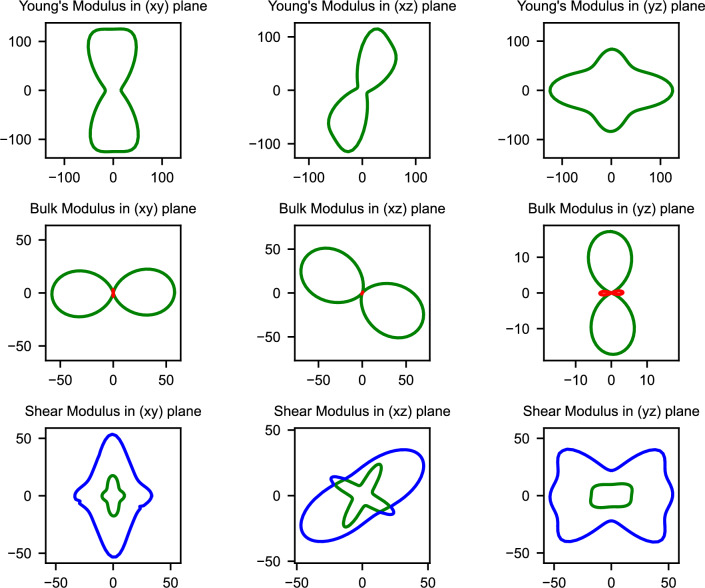
Spatial dependence of Young’s modulus (E), bulk modulus (K), and shear modulus (G) for GeP-mono. The pronounced anisotropy reflects the layered monoclinic structure with highly directional mechanical properties.

**Fig. 3:**
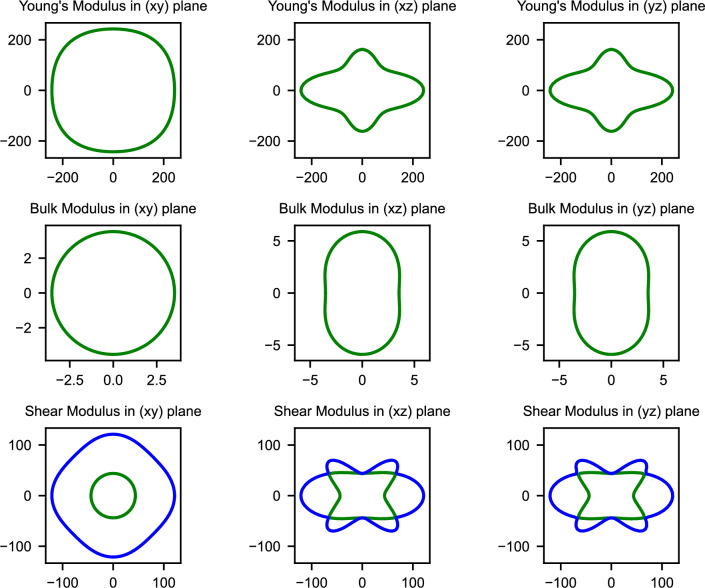
Spatial dependence of Young’s modulus (E), bulk modulus (K), and shear modulus (G) for GeP-tetra. The tetragonal symmetry results in relatively isotropic behavior within the ab-plane with moderate c-axis anisotropy.

**Fig. 4:**
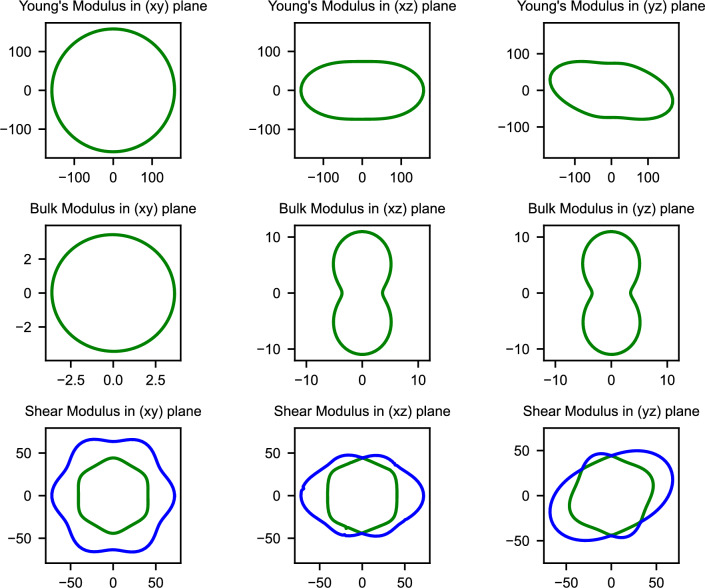
Spatial dependence of Young’s modulus (E), bulk modulus (K), and shear modulus (G) for GeP3. The rhombohedral symmetry leads to intermediate anisotropy with smoother directional variations.

Understanding the directional dependence of mechanical properties becomes particularly crucial when considering the anisotropic stress conditions experienced during battery cycling, where lithiation-induced volume changes can generate complex stress states. The spatial dependence of elastic moduli, illustrated in Figs. 2, 3, and 4, reveals how mechanical properties vary with crystallographic direction for the three mechanically stable structures.

GeP-mono demonstrates the most extreme elastic anisotropy among the investigated structures, with Young’s modulus exhibiting dramatic variations across crystallographic directions as visualized in Fig. 2. This pronounced directional dependence directly reflects the layered monoclinic architecture and indicates that mechanical response during lithiation will be strongly orientation-dependent.

In contrast, GeP-tetra exhibits relatively isotropic behavior within the ab-plane due to its tetragonal symmetry, with more pronounced anisotropy emerging along the z-axis direction as shown in Fig. 3. The high and relatively uniform in-plane elastic moduli demonstrate exceptional resistance to deformation within the crystallographic layers. This moderate anisotropy, when combined with the exceptionally high absolute modulus values, suggests more predictable and controllable mechanical behavior compared to the highly variable response of GeP-mono.

Completing the anisotropy spectrum, $\hbox {GeP}_{3}$ demonstrates intermediate behavior where the rhombohedral symmetry produces more uniform in-plane properties than GeP-mono while maintaining less uniformity than GeP-tetra, as illustrated in Fig. 4. The spatial distribution reveals smoother transitions between maximum and minimum elastic moduli values, indicating more gradual and predictable directional variations in mechanical properties. This moderate anisotropy potentially provides an optimal balance between structural stability and effective stress distribution during the volume changes associated with electrochemical cycling.

The mechanical stability analysis confirms that all stable compounds satisfy the stability criteria for their respective crystal systems, establishing their fundamental thermodynamic viability. The Pugh’s ratios (K/G) of 1.20 for GeP-mono, 1.06 for GeP-tetra, and 1.17 for $\hbox {GeP}_{3}$ all fall below the 1.75 threshold that distinguishes brittle from ductile behavior according to Pugh’s criterion^41^, indicating inherently brittle mechanical response typical of covalently bonded semiconductors. This brittleness suggests potential susceptibility to fracture under large deformations, requiring careful consideration for battery electrode design where mechanical integrity during cycling is paramount.

Further insight into the bonding character emerges from Cauchy pressure analysis (C$_{12}$ - C$_{44}$), where all stable phases exhibit negative values indicating predominantly covalent bonding character: GeP-mono (−5.5 GPa), GeP-tetra (−93.8 GPa), and $\hbox {GeP}_{3}$ (−23.3 GPa). The exceptionally negative Cauchy pressure in GeP-tetra reflects the strongest covalent bonding, consistent with its exceptional stiffness, while the intermediate values in $\hbox {GeP}_{3}$ and GeP-mono suggest varying degrees of covalent character that correlate with their respective mechanical properties.

Quantitative assessment of elastic anisotropy through the universal anisotropy index (A$_U$, where A$_U$ = 0 represents perfect isotropy) reveals a clear hierarchy among the stable phases. GeP-mono demonstrates extremely high anisotropy (A$_U$ = 7.90) reflecting its layered monoclinic structure with dramatically different mechanical properties along different crystallographic directions. GeP-tetra exhibits intermediate anisotropy (A$_U$ = 1.20) due to its tetragonal symmetry with pronounced c-axis differences, while $\hbox {GeP}_{3}$ shows the lowest anisotropy (A$_U$ = 0.77) approaching more isotropic behavior. These anisotropy differences carry crucial implications for battery performance, as the extreme directional dependence in GeP-mono could generate preferential expansion and contraction during lithiation that leads to mechanical stress concentration and capacity degradation. The moderate anisotropy of GeP-tetra provides intermediate behavior, while the more uniform mechanical response of $\hbox {GeP}_{3}$ suggests more predictable volume changes that could enhance cycling stability.

The mechanical property analysis provides direct insights into electrochemical cycling behavior and structural degradation mechanisms. During lithiation, phosphide anodes typically undergo substantial volume expansion (100–300%), generating significant internal stresses that can lead to particle fracture, pulverization, and loss of electrical connectivity. GeP-mono’s moderate bulk modulus (32.1 GPa) enables stress relaxation and volume accommodation through elastic deformation, potentially extending cycle life by maintaining particle integrity. However, its pronounced elastic anisotropy (A$_U$ = 7.90) indicates directional mechanical response where crack propagation will preferentially occur along weak crystallographic planes. This anisotropic behavior could be exploited through particle orientation control or electrode architecture design to minimize stress concentration along vulnerable directions. In contrast, GeP-tetra’s exceptionally high bulk modulus (79.4 GPa) creates substantial resistance to volume changes, increasing the risk of particle fracture during repeated lithiation/delithiation cycles. The Pugh’s ratio analysis confirms that all stable phases exhibit brittle behavior (K/G < 1.75), indicating limited plastic deformation capability. This brittleness necessitates careful electrode engineering strategies such as nanostructuring, carbon coating, or composite formation to mitigate mechanical degradation. GeP-cubic’s mechanical instability, evidenced by negative shear moduli that violate Born stability criteria, predicts catastrophic structural collapse under any applied stress, definitively eliminating this polymorph from practical battery applications regardless of other potentially favorable properties. The intermediate mechanical properties of $\hbox {GeP}_{3}$–combining substantial stiffness (61.0 GPa bulk modulus) with relatively low anisotropy (A$_U$ = 0.77)–suggest an optimal balance between structural integrity and volume accommodation capability, potentially offering superior cycling stability compared to the GeP polymorphs.

### Electronic properties

The electronic structure of GeP and $\hbox {GeP}_{3}$ provides fundamental insights into their electrical conductivity, charge transport mechanisms, and electrochemical activity, all of which directly influence their performance as battery electrode materials. Through comprehensive density of states (DOS) and band structure calculations across all polymorphs (GeP-mono, GeP-tetra, GeP-cubic, and $\hbox {GeP}_{3}$), we establish the electronic foundations that govern their battery-relevant properties and identify key differences arising from structural and compositional variations.

The GeP polymorphs and $\hbox {GeP}_{3}$ exhibit diverse electronic behaviors ranging from semiconducting to metallic character. GeP-mono maintains semiconducting behavior with a PBE band gap of 0.45 eV (VBM: 5.79 eV, CBM: 6.24 eV), which increases to 0.77 eV with the TB-mBJ functional (VBM: 5.83 eV, CBM: 6.60 eV), approaching experimental values. In contrast, GeP-tetra, GeP-cubic, and $\hbox {GeP}_{3}$ demonstrate metallic behavior with overlapping valence and conduction bands. TB-mBJ calculations confirm the metallic nature of GeP-cubic (Fermi level: 6.17 eV) and $\hbox {GeP}_{3}$ (Fermi level: 7.01 eV), validating that their metallicity is an intrinsic property rather than a DFT artifact. The semiconducting character of GeP-mono represents typical behavior for layered Group IV-V compounds^5^.

To validate the robustness of our electronic structure predictions and address the well-known band gap underestimation of standard DFT functionals, we performed additional calculations using the Tran-Blaha modified Becke-Johnson (TB-mBJ) meta-GGA functional for all phases. Table 4 presents a comprehensive comparison of band gap values obtained from different computational methods alongside experimental and literature values. The TB-mBJ functional yields a band gap of 0.77 eV for GeP-mono, representing a substantial improvement over the PBE value (0.45 eV) and approaching the experimental value (0.90 eV) and HSE06 prediction (0.94 eV). This improved agreement validates that the semiconducting nature of GeP-mono is not an artifact of functional choice but rather an intrinsic electronic property. Importantly, TB-mBJ calculations confirm that GeP-cubic and $\hbox {GeP}_{3}$ remain metallic with no band gap opening, demonstrating that their metallic character is robust across different levels of theory and represents a fundamental electronic property of these crystal structures.

**Table 4:** Band gap comparison for GeP and $\hbox {GeP}_{3}$ across different dimensionalities and computational methods. TB-mBJ calculations confirm that GeP-cubic and $\hbox {GeP}_{3}$ remain metallic, validating the robustness of their electronic properties across different functionals.

Material	Method	Band Gap (eV)	Type
This work – Bulk phases			
GeP-mono	PBE-D2	0.45	Indirect
GeP-mono	TB-mBJ	0.77	Indirect
GeP-tetra	PBE-D2	0 (metallic)	–
GeP-cubic	PBE-D2	0 (metallic)	–
GeP-cubic	TB-mBJ	0 (metallic)	–
$\hbox {GeP}_{3}$	PBE-D2	0 (metallic)	–
$\hbox {GeP}_{3}$	TB-mBJ	0 (metallic)	–
Literature – Bulk GeP (monoclinic)			
Bulk	Experimental^26^	0.90	Indirect
Bulk	HSE06^5^	0.94	Indirect
Literature – Multilayer GeP			
Monolayer	HSE06^5^	2.31	Indirect
Monolayer	Experimental^5^	2.30	–
Bilayer	HSE06^5^	1.66	–
Trilayer	HSE06^5^	1.47	–
Tetralayer	HSE06^5^	1.40	–
Literature –$\hbox {GeP}_{3}$multilayers			
Monolayer	HSE06^32^	0.55	Indirect
Monolayer	PBE^32^	0.27	Indirect
Bilayer	HSE06^32^	0.43	Indirect
Trilayer	HSE06^32^	0 (metallic)	–
Bulk	HSE06^32^	0 (metallic)	–

Table 4 reveals a pronounced thickness-dependent band gap evolution for both GeP and $\hbox {GeP}_{3}$, demonstrating the critical role of dimensionality in determining electronic properties. For GeP, the band gap decreases systematically from 2.31 eV in the monolayer to 0.90 eV in the bulk phase, representing a 61% reduction driven by enhanced interlayer interactions and quantum confinement effects. This trend follows the expected behavior for layered materials where reduced dimensionality increases quantum confinement and widens the band gap. The experimental monolayer value (2.30 eV) shows excellent agreement with HSE06 calculations (2.31 eV), validating the computational predictions. For $\hbox {GeP}_{3}$, an even more dramatic transition occurs: the material evolves from semiconducting in monolayer (0.55 eV) and bilayer (0.43 eV) forms to metallic behavior in trilayer and bulk phases. This semiconductor-to-metal transition at the trilayer thickness represents a critical dimensional threshold where interlayer coupling becomes sufficiently strong to close the band gap entirely. Our bulk calculations for both GeP-mono (0.77 eV with TB-mBJ) and $\hbox {GeP}_{3}$ (metallic) are consistent with these literature trends, confirming that the dimensional evolution of electronic properties follows physically reasonable behavior from 2D to 3D systems.

The phosphorus-rich $\hbox {GeP}_{3}$ composition exhibits metallic behavior despite its enhanced phosphorus content that introduces greater p-orbital character into the electronic states, indicating that structural factors dominate over compositional effects in determining the electronic properties. While the indirect band gap nature of GeP-mono suggests relatively weak optical transitions, this characteristic does not significantly constrain battery application potential where electronic conductivity and electrochemical activity take precedence over optical properties. Remarkably, the electronic behavior varies dramatically across polymorphs: GeP-mono maintains moderate semiconducting character (0.450 eV band gap), while GeP-tetra, GeP-cubic, and $\hbox {GeP}_{3}$ exhibit metallic behavior with no band gap, providing excellent intrinsic electronic conductivity that eliminates the need for conductivity enhancement strategies typically required for semiconductor electrodes.

**Fig. 5:**
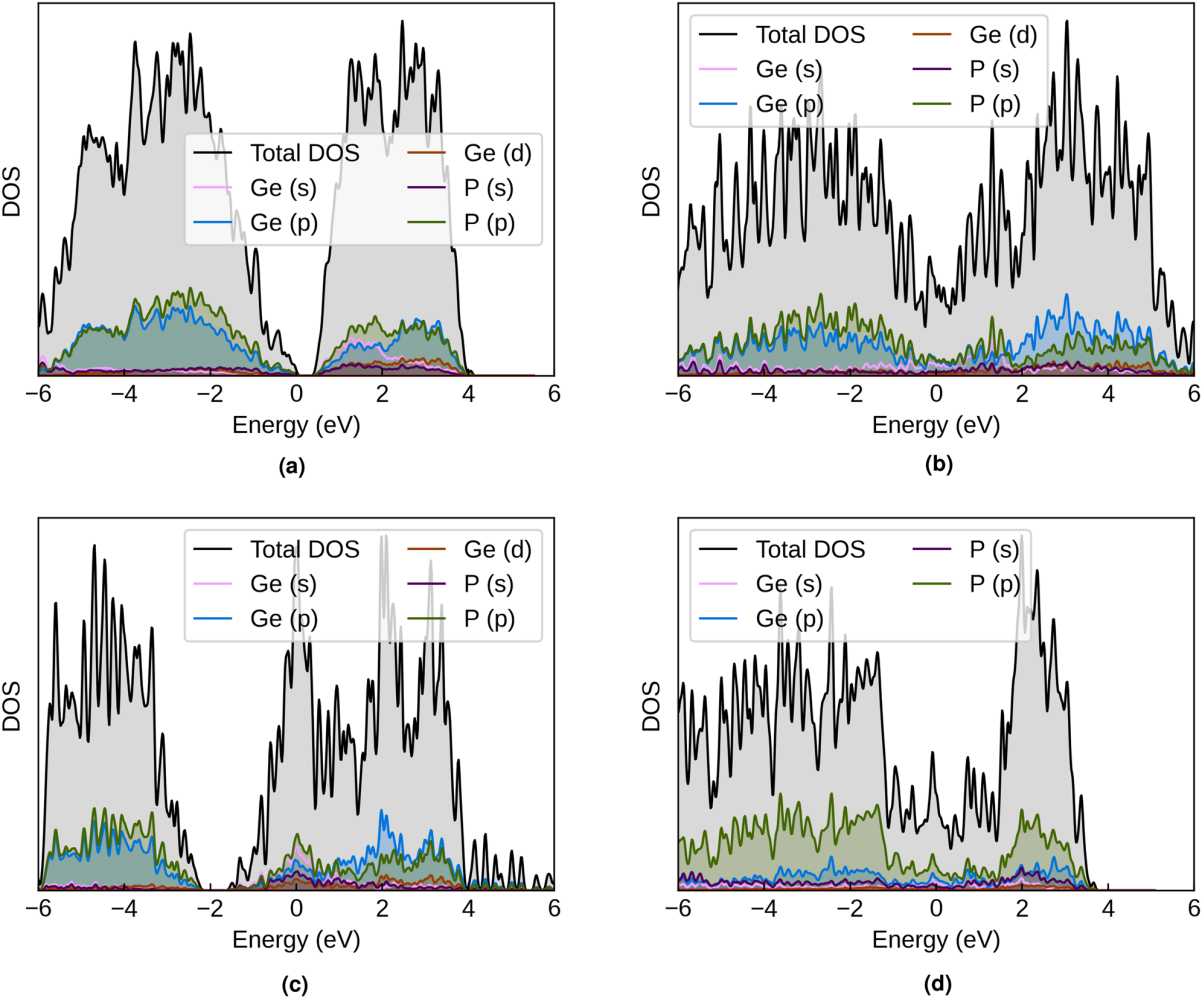
Density of states (DOS) for GeP polymorphs and $\hbox {GeP}_{3}$. The plots show total DOS (black line) and projected DOS contributions from different elements and orbitals. The Fermi level is set to zero energy. The calculations reveal that GeP-mono exhibits semiconducting behavior with a 0.450 eV band gap, while GeP-tetra, GeP-cubic, and $\hbox {GeP}_{3}$ display metallic behavior with no band gap.

The comprehensive density of states analysis provides detailed electronic structure insights that illuminate the bonding characteristics and electrochemical activity potential of both compounds. Figure 5a, b, c, and d present the calculated total and projected DOS for all investigated polymorphs, revealing distinct electronic signatures that directly reflect the structural and compositional differences previously established.

The orbital composition analysis reveals that GeP-mono’s valence band consists primarily of P 3p orbitals with substantial Ge 4p contributions, establishing strong covalent bonding between germanium and phosphorus atoms. This valence band extends approximately 6 eV below the Fermi level, with the highest occupied states displaying mixed Ge-P character that facilitates charge transport and electrochemical activity. The conduction band manifests predominantly Ge 4 s and 4p orbital character, with the lowest unoccupied states positioned about 0.45 eV above the Fermi level, creating accessible pathways for electron injection during battery operation.

$\hbox {GeP}_{3}$ demonstrates similar overall DOS characteristics but with significantly enhanced P 3p contributions reflecting its higher phosphorus content, resulting in more complex valence band structure that incorporates increased P-P interactions throughout the crystal framework. While the conduction band retains substantial Ge character, it exhibits enhanced hybridization with P orbitals compared to GeP-mono, creating more delocalized electronic states that could facilitate improved charge transport. This electronic structure evolution directly correlates with the enhanced mechanical properties previously demonstrated, as the increased P-P bonding network establishes the robust three-dimensional covalent framework responsible for the superior elastic moduli.

The diverse electronic properties have direct implications for charge transport and battery electrode performance. GeP-mono’s semiconducting nature with a band gap of 0.77 eV (TB-mBJ, approaching the experimental value of 0.90 eV) results in thermally-activated conductivity at room temperature. Using the relation $n \propto \exp (-E_g/2k_BT)$, the intrinsic carrier concentration at 298 K is estimated to be on the order of 10$^{13}$−10$^{14}$ cm$^{-3}$. This relatively low intrinsic conductivity necessitates electrode engineering strategies such as carbon coating, conductive additive incorporation, or strategic doping to achieve adequate electronic percolation for high-rate battery applications. The requirement for conductivity enhancement is more stringent than initially predicted by PBE calculations due to the larger, more accurate band gap. In contrast, the metallic phases (GeP-tetra, GeP-cubic, $\hbox {GeP}_{3}$) exhibit high density of states at the Fermi level with true band overlap confirmed by TB-mBJ calculations, indicating abundant charge carriers and excellent intrinsic electronic conductivity comparable to conventional metallic electrode materials. This eliminates conductivity limitations and enables facile electron transport throughout the electrode without extensive conductive matrix requirements, potentially allowing higher rate capabilities and simplified electrode architectures.

To complement these DOS analyses, comprehensive partial charge density visualizations are provided in the Supporting Information (Section S3), revealing the spatial distribution of electronic states near the Fermi level. For $\hbox {GeP}_{3}$, these charge density maps directly visualize the highly delocalized electron distribution responsible for metallic conductivity, showing strong covalent Ge-P bonding within layers and relatively uniform in-plane charge distribution that correlates with the moderate elastic anisotropy and isotropic electronic transport. The charge density patterns for all phases provide direct electronic structure evidence for the bonding characteristics and structure-property relationships discussed throughout this work.

### Thermodynamic properties

Thermodynamic properties provide critical insights into thermal stability and temperature-dependent performance for battery electrode applications. Using Debye model analysis based on calculated elastic constants, we characterize the thermal response of all mechanically stable phases.

**Fig. 6:**
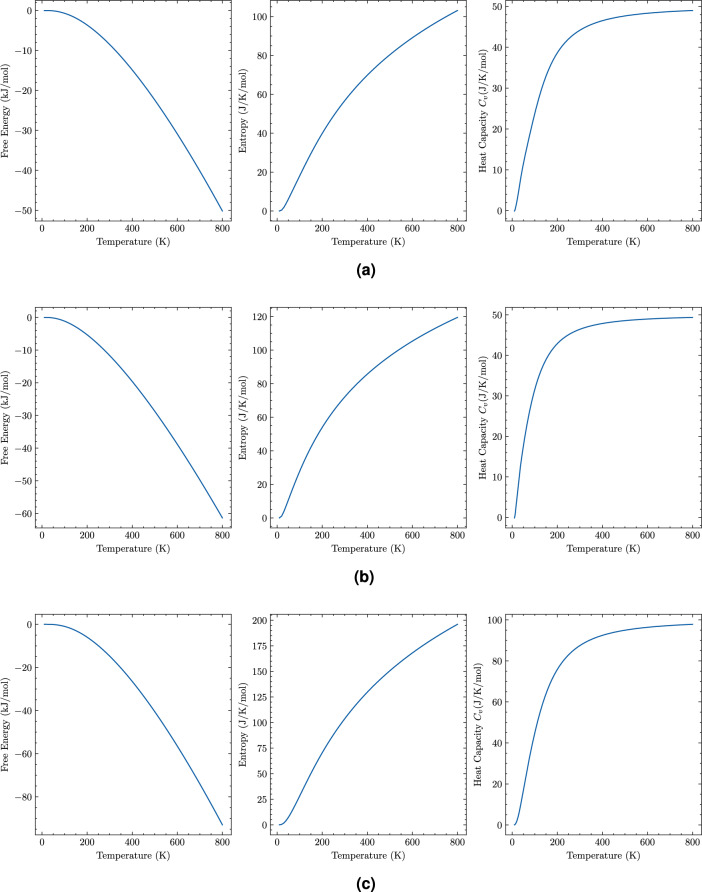
Thermodynamic curves (heat capacity C$_V$, entropy S, Helmholtz free energy F) computed from elastic-based Debye analysis. GeP-cubic is excluded due to mechanical instability.

Temperature-dependent heat capacity analysis (0–800 K) shows systematic progression at 298 K: GeP-mono (44.1 J/(mol$\cdot$K)), GeP-tetra (46.4 J/(mol$\cdot$K)), GeP-cubic (47.1 J/(mol$\cdot$K)), and $\hbox {GeP}_{3}$ (87.3 J/(mol$\cdot$K)). The dramatic increase in $\hbox {GeP}_{3}$ reflects both larger atomic content and enhanced P-P interactions. Heat capacity curves exhibit characteristic crystalline behavior with C$_V$ following T³ dependence at low temperatures before approaching classical limits. Figure 6 presents complete thermodynamic characterization through temperature-resolved curves of heat capacity (C$_V$), entropy (S), and Helmholtz free energy (F) for all mechanically stable structures, confirming $\hbox {GeP}_{3}$’s superior thermal characteristics.

**Table 5:** Thermochemical properties at 298 K calculated from elastic-based Debye analysis.

Phase	*S* (J mol$^{-1}$ K$^{-1}$)	$C_V$ (J mol$^{-1}$ K$^{-1}$)
GeP-mono	56.5	44.1
GeP-tetra	71.8	46.4
GeP-cubic	74.9	47.1
GeP3	103.2	87.3

Entropy analysis reveals systematic phase progression from GeP-mono (56.5 J/(mol$\cdot$K)) to $\hbox {GeP}_{3}$ (103.2 J/(mol$\cdot$K)), reflecting increased vibrational mode density. Helmholtz free energy calculations show negative values relative to constituent elements, confirming thermodynamic stability. Phase stability analysis establishes both compounds as stable configurations with negative formation energies, while $\hbox {GeP}_{3}$’s elevated Debye temperature indicates reduced sensitivity of elastic properties to temperature variations.

The presented thermochemical properties rely entirely on theoretical calculations due to limited experimental data for these specific polymorphs. However, the theoretical framework demonstrates consistency with analogous Group IV-V compounds and provides reliable predictions within DFT accuracy limits.

The Debye model approach, while computationally efficient and providing valuable comparative insights, has inherent limitations that should be acknowledged. The harmonic approximation underlying the Debye model becomes increasingly inaccurate at elevated temperatures, typically above 0.5–0.7.5.7 $\Theta _D$. For our materials, this corresponds to temperatures above approximately 150–320 K, suggesting that predictions at higher temperatures should be interpreted as semi-quantitative trends rather than quantitatively precise values. At temperatures relevant to battery thermal runaway scenarios (above 400 K), anharmonic effects including thermal expansion and phonon-phonon interactions become significant, and full phonon dispersion calculations would be required for quantitative accuracy. Additionally, the elastic-based Debye approach assumes isotropic sound velocities, which represents an approximation for our anisotropic layered structures. Despite these limitations, the Debye model provides reliable comparative analysis for room-temperature properties and establishes clear trends among the different phases.

The thermodynamic parameters calculated here have direct practical significance for battery electrode applications. Heat capacity (C$_V$) determines thermal buffering capability during exothermic lithiation reactions and ohmic heating during charge/discharge cycles. The substantially higher heat capacity of $\hbox {GeP}_{3}$ (87.3 J/(mol$\cdot$K)) compared to GeP polymorphs (44–47 J/(mol$\cdot$K)) provides superior thermal management, reducing temperature spikes that accelerate degradation reactions and improving safety margins during fast-charging operations. Entropy (S) reflects the density of vibrational states and configurational freedom, with higher entropy correlating with enhanced accommodation of structural disorder during cycling and potentially facilitating lithium diffusion through the lattice. The Debye temperature ($\Theta _D$) serves as an indicator of interatomic bonding strength and thermal stability. $\hbox {GeP}_{3}$’s higher Debye temperature (459 K) compared to GeP-mono (302 K) indicates stronger bonding and greater resistance to thermally-induced structural degradation, particularly important for automotive applications requiring operation across wide temperature ranges (−40 to +85${\circ }$C) and providing enhanced safety margins during thermal abuse conditions. Future investigations incorporating anharmonic phonon effects could enhance understanding of temperature-dependent behavior under extreme battery operating conditions.

### Discussion

The integrated analysis of GeP polymorphs and $\hbox {GeP}_{3}$ reveals critical structure-property relationships governing their battery anode property. The polymorphic diversity demonstrates how crystal structure fundamentally alters material behavior despite identical composition, with mechanical stability emerging as the primary determinant for practical applications. The stability hierarchy establishes GeP-mono as the optimal GeP polymorph, combining mechanical robustness with layered architecture that accommodates lithium insertion-induced volume expansion. Its moderate bulk modulus (32.1 GPa) enables stress relaxation during the substantial volume changes (100–300%) typical of phosphide anodes, while its pronounced elastic anisotropy (A$_U$ = 7.90) indicates directional mechanical response that can be exploited through particle orientation control to minimize crack propagation along vulnerable crystallographic planes. GeP-tetra’s exceptional bulk modulus (79.4 GPa) provides superior structural strength but creates significant risk of particle fracture during repeated cycling due to limited volume accommodation capability. The Pugh’s ratio analysis (K/G < 1.75 for all stable phases) confirms inherently brittle behavior, necessitating careful electrode engineering strategies such as nanostructuring or carbon coating to mitigate mechanical degradation. GeP-cubic’s mechanical instability, evidenced by negative shear moduli that violate Born stability criteria, predicts catastrophic structural collapse under electrochemical stress, definitively eliminating it from practical consideration.

The electronic structure evolution reveals diverse behaviors ranging from semiconducting (GeP-mono, 0.45 eV band gap) to metallic (GeP-tetra, GeP-cubic, $\hbox {GeP}_{3}$). The metallic polymorphs provide excellent intrinsic conductivity, eliminating traditional semiconductor limitations and reducing electrode engineering requirements. This electronic diversity offers opportunities for tailored applications based on conductivity needs. $\hbox {GeP}_{3}$ emerges as mechanically superior across all metrics, achieving optimal balance between strength (61 GPa bulk modulus) and cycling accommodation capability through its relatively low elastic anisotropy (A$_U$ = 0.77) that ensures more uniform and predictable volume changes. Its superior thermodynamic characteristics–including higher Debye temperature (459 K vs. 302 K for GeP-mono) indicating stronger interatomic bonding, substantially higher heat capacity (87.3 J/(mol$\cdot$K)) providing enhanced thermal buffering during fast charging and improved safety margins, and elevated entropy reflecting greater structural flexibility–position it as the preferred choice for high-temperature and safety-critical applications, despite reduced theoretical capacity compared to GeP alternatives.

Electrode design strategies must accommodate distinct mechanical characteristics and address the brittle nature of these materials. GeP-mono’s anisotropic properties benefit from controlled particle alignment or layered architectures that manage directional expansion and minimize stress concentration along weak crystallographic planes, though its brittleness (K/G = 1.20) requires nanostructuring to prevent catastrophic fracture during volume expansion. GeP-tetra requires nanoscale particles to prevent fracture while leveraging high stiffness for structural integrity, with its exceptionally brittle character (K/G = 1.06) demanding careful particle size control and potentially carbon coating for mechanical reinforcement. $\hbox {GeP}_{3}$’s balanced mechanical behavior–combining substantial stiffness with low anisotropy and moderate brittleness (K/G = 1.17)–enables more conventional electrode architectures with enhanced cycling stability, while its superior thermal properties provide additional safety margins during fast charging and high-temperature operation.

While comprehensive mechanical property data for bulk $\hbox {SnP}_{3}$ and GeAs phases are scarce in the literature, 2D monolayer studies provide valuable qualitative context for understanding broader trends in Group IV-V battery anode materials. Ghosh et al.^42^ reported that monolayer $\hbox {SnP}_{3}$ exhibits nearly isotropic elastic behavior ($C \approx$ 31 N/m), qualitatively consistent with the trend observed for monolayer $\hbox {GeP}_{3}$ ($C \approx$ 22 N/m^32^), suggesting that P-rich triphosphide stoichiometries promote mechanically uniform frameworks. Similarly, monolayer GeAs studies^43,44^ report moderate mechanical anisotropy (Young’s modulus ratio $\sim$1.2:1), qualitatively intermediate between our bulk phases. However, direct quantitative comparison between these 2D monolayer results and our bulk materials requires caution due to substantial dimensionality effects–mechanical properties can differ significantly between 2D monolayers and bulk materials due to different bonding environments, quantum confinement, and surface effects. Our systematic comparison across bulk GeP polymorphs and bulk $\hbox {GeP}_{3}$, revealing anisotropy indices spanning nearly an order of magnitude (0.77–7.90), provides the most relevant design guidelines for bulk battery anode applications and demonstrates that crystal structure is the primary determinant of mechanical anisotropy in these materials.

Future research should prioritize experimental validation through in-situ mechanical testing during electrochemical cycling, investigation of lithiation mechanisms and volume changes, detailed Li/Na diffusion pathway analysis building upon existing computational studies^5,11^, and exploration of composite designs. The intrinsic structural, mechanical, electronic, and thermodynamic properties characterized in this work provide essential foundations upon which such intercalation and diffusion studies can be developed. The polymorphic diversity suggests opportunities for phase engineering approaches to optimize properties through controlled synthesis or transformation management. This investigation establishes mechanical properties as primary determinants of battery performance, providing quantitative foundations for rational anode materials design that balances capacity, stability, and safety for next-generation electrochemical energy storage.

## Conclusions

We performed a comprehensive first-principles investigation of the structural, mechanical, electronic, and thermodynamic properties of GeP polymorphs (monoclinic C2/m, tetragonal I4mm, and cubic F-43m) and $\hbox {GeP}_{3}$ (R-3m) to assess their suitability as ion battery anode materials. Our investigation reveals that polymorphism fundamentally governs GeP properties, with the three polymorphs exhibiting markedly different mechanical responses and electronic structures despite identical composition. GeP-mono demonstrates mechanical stability with pronounced elastic anisotropy, while GeP-tetra shows exceptional stiffness with isotropic in-plane behavior. GeP-cubic proves mechanically unstable and unsuitable for practical applications. Electronic structure analysis reveals diverse behaviors from semiconducting (GeP-mono) to metallic (GeP-tetra, GeP-cubic, $\hbox {GeP}_{3}$), providing excellent intrinsic conductivity for most phases.

The distinct characteristics suggest different optimization strategies for battery electrode design. Among GeP structures, GeP-mono presents optimal balance between compliance and stability, accommodating lithiation-induced volume changes through anisotropic elastic response and preferred particle orientations. GeP-tetra exhibits exceptional structural integrity but requires nanoscale particle control to prevent mechanical fracture during volume expansion. $\hbox {GeP}_{3}$ emerges as particularly attractive, offering superior thermodynamic and mechanical stability despite lower theoretical capacity. Its higher Debye temperature, enhanced entropy and heat capacity, and stronger interatomic bonding position it as the preferred choice for applications prioritizing electrode durability and operational safety over maximum gravimetric capacity.

These findings establish clear design implications that GeP-mono suits high-capacity applications with architecture-assisted strain accommodation, GeP-tetra shows promise for stiffness-critical designs with nanoscale particles, and $\hbox {GeP}_{3}$ excels in robust, thermally stable electrode applications.

Future experimental research should prioritize mechanical characterization of the specific phases investigated. Theoretical predictions, particularly thermodynamic properties lacking experimental data, require validation through targeted synthesis of GeP-mono, GeP-tetra, and $\hbox {GeP}_{3}$ phases. Additionally, the role of defects might also be important for the properties of those phases. Defects, such as vacancies, interstitials, and phase boundaries, can significantly influence the mechanical, electronic, and electrochemical behavior of materials. Future research should investigate the effects of defects on the performance of these anode materials, including their impact on mechanical stability, electronic conductivity, and cycling durability.

Our findings highlight the crucial role of polymorphism and mechanical anisotropy in dictating electrode performance, providing quantitative guidance for materials selection, electrode architecture, and cycling strategies while establishing foundations for experimental validation and phase-engineering approaches.

## Supplementary Information

Supplementary Information.

## Data Availability

All computational input files to support the findings of this study are provided in the Supporting Information. Additional datasets generated during the current study are available from the corresponding author on reasonable request.
