# Supplementary material for: Mechanical stability and thermodynamic properties of GeP and [image] as battery anode materials from first principles
Source: Sci Rep. 2026 Jan 23;16:6058. doi: 10.1038/s41598-026-36336-1 (PMC12901247; doi:10.1038/s41598-026-36336-1)
Supplement: Supplementary file 1 — Supplementary Information. [file 41598_2026_36336_MOESM1_ESM.pdf]

Supporting Information: Mechanical stability and thermodynamic properties of GeP and GeP_3 as battery anode materials from first principles

Duc Toan Truong,[†,‡] Nguyen-Hieu Hoang,[¶] Chi M. Phan,[§] An-Giang Nguyen,[*,‖] and Thuat T. Trinh[*,⊥]

†*Laboratory for Chemical Computation and Modeling, Institute for Computational Science and Artificial Intelligence, Van Lang University, Ho Chi Minh City, 70000, Vietnam*

‡*Faculty of Applied Technology, Van Lang School of Technology, Van Lang University, Ho Chi Minh City, 70000, Vietnam*

¶*Department of Materials and Nanotechnology, SINTEF Industry, Trondheim, NO-7034, Norway*

§*Discipline of Chemical Engineering, WASM MECE, Curtin University, Perth, WA 6045, Australia*

‖*College of Engineering and Computer Science, VinUniversity, Hanoi 100000, Vietnam*

⊥*Porelab, Department of Chemistry, Norwegian University of Science and Technology, Høgskoleringen 5, 7491-Trondheim, Norway*

E-mail: giang.na@vinuni.edu.vn; thuat.trinh@ntnu.no

This Supplementary Information provides comprehensive details supporting the main manuscript, including: (i) systematic convergence tests for computational parameters, (ii)

validation of the exchange-correlation functional choice through comparison with experimental data, (iii) detailed partial charge density analysis revealing bonding characteristics, and (iv) phonon dispersion calculations confirming dynamic stability.

S1. Convergence Tests for Computational Parameters

To ensure the reliability and accuracy of our DFT calculations, we performed systematic convergence tests for the plane-wave energy cutoff (ENCUT) and k-point mesh density for GeP3. These tests are essential for establishing appropriate computational parameters that balance accuracy and computational efficiency.

ENCUT Convergence

Figure S1 shows the convergence of total energy per atom and stress tensor norm as a function of plane-wave energy cutoff (ENCUT) for GeP3 using a fixed 4×4×2 k-point mesh. The energy difference relative to ENCUT = 850 eV decreases from 3.3 meV/atom at 300 eV to 0.068 meV/atom at 600 eV, while the stress tensor norm difference reduces to 0.08 GPa at 600 eV. We selected ENCUT = 600 eV for all production calculations, ensuring convergence within $\leq$ 0.1 meV/atom for energy and $\leq$ 0.1 GPa for stress.

K-Point Mesh Convergence

Figure S2 presents the convergence of total energy per atom and stress tensor norm as a function of k-point spacing (in units of $2\pi/a$) for GeP3 at fixed ENCUT = 600 eV. The reference calculation uses a 9×9×5 mesh (spacing = 0.0186 × $2\pi/a$). The energy difference increases from 0.5 meV/atom at 0.020 × $2\pi/a$ to 8.1 meV/atom at 0.053 × $2\pi/a$, while stress tensor differences remain below 1.3 GPa. We selected a 4×4×2 mesh (spacing ≈ 0.043 × $2\pi/a$) for production calculations, providing good balance between accuracy and efficiency with 3.5 meV/atom energy difference from the densest mesh.

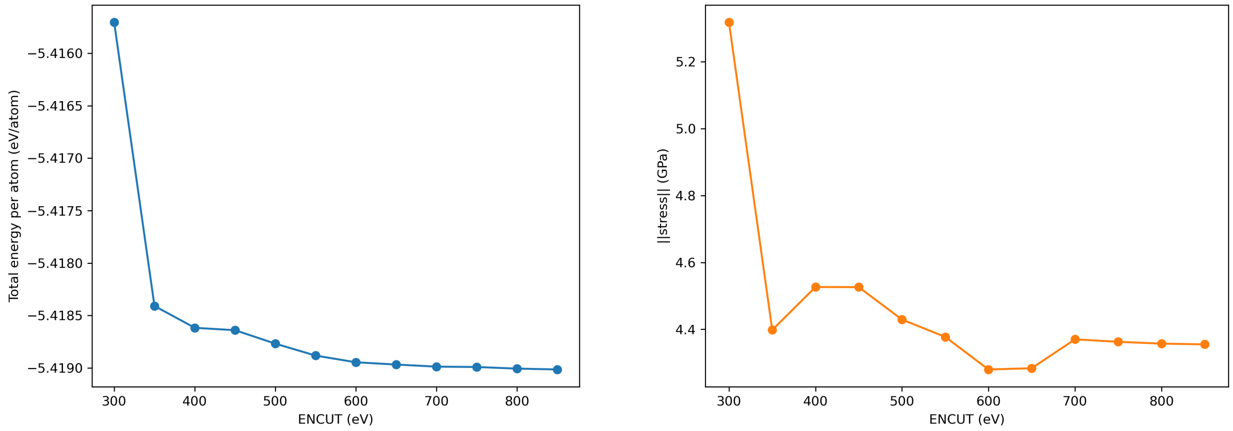

(a) Total energy per atom

(b) Stress tensor norm

Figure S1: ENCUT convergence tests for GeP3. (a) Total energy per atom as a function of ENCUT. (b) Stress tensor norm as a function of ENCUT. The reference calculation uses ENCUT = 850 eV. All calculations were performed with a 4×4×2 k-point mesh.

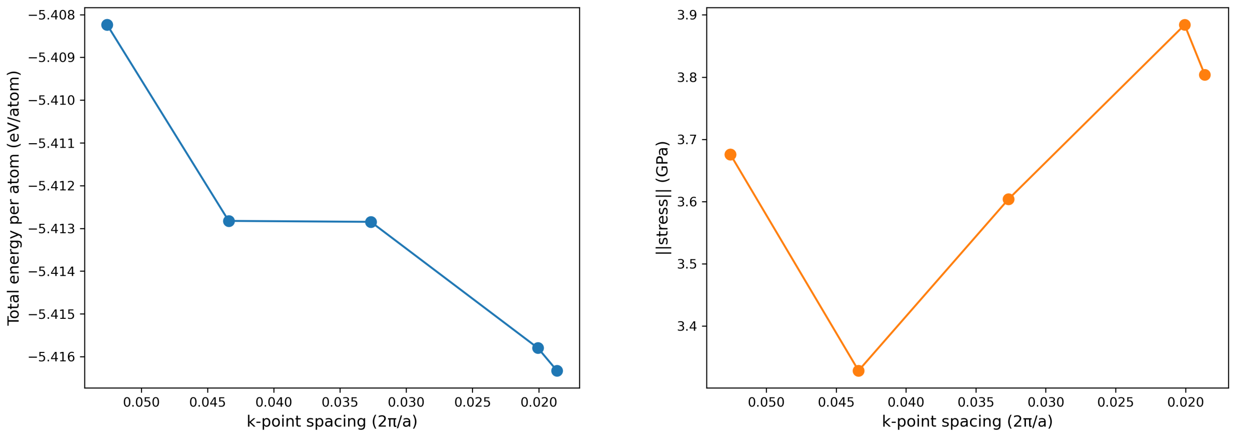

(a) Total energy per atom

(b) Stress tensor norm

Figure S2: K-point mesh convergence tests for GeP3. (a) Total energy per atom as a function of k-point spacing (in units of $2\pi/a$). (b) Stress tensor norm as a function of k-point spacing. The reference calculation uses a 9×9×5 k-point mesh (spacing = $0.0186 \times 2\pi/a$). All calculations were performed with ENCUT = 600 eV. The x-axis is reversed so that smaller spacing (better convergence) appears on the right.

S2. Comparison of DFT Methods for Structural Parameters

To assess the performance of different exchange-correlation functionals and van der Waals corrections, we performed structural optimizations using four different DFT methods: PBE, PBE-D3, optb88-vdw, and PBE-D2 (the main method used in this work). Tables S1 and S2 present comparisons of calculated lattice parameters with experimental values for GeP3[S1] and GeP polymorphs,[S2] respectively. This systematic comparison provides the rationale for selecting PBE-D2 as the primary computational method throughout our study.

Method Selection Rationale

PBE-D2 was selected as the main computational method because it provides the best balance of accuracy across all phases studied. While PBE-D3 shows superior performance for GeP3 and GeP-tetra, PBE-D2 excels for GeP-mono (the most stable experimental phase with average deviation of 0.29%) and maintains reasonable accuracy for other polymorphs (average volume deviations: 2.30% for GeP3, 0.52% for GeP-tetra, 6.31% for GeP-cubic). The consistent performance of PBE-D2 across diverse structural types makes it most suitable for comparative studies of GeP-based materials. Furthermore, PBE-D2's balanced description of both covalent bonding and van der Waals interactions is critical for predicting mechanical and thermodynamic properties relevant to battery anode applications.

Table S1: Comparison of lattice parameters for GeP3 calculated using different DFT methods. Experimental values from Ref.[S1] Deviations: $\Delta = 100 \times (X_{\text{calc}} - X_{\text{exp}})/X_{\text{exp}}$.

Method	a (Å)		c (Å)		V (Å^3)	
	Calc	Δ (%)	Calc	Δ (%)	Calc	Δ (%)
Experimental[1]	7.050	–	9.932	–	427.5	–
PBE-D2 (main)	7.075	0.36	9.633	−3.01	417.7	−2.30
PBE	7.114	0.91	9.995	0.64	438.1	2.48
PBE-D3	7.075	0.35	9.788	−1.44	424.3	−0.75
optb88-vdw	7.145	1.34	10.117	1.87	447.3	4.63

Table S2: Comparison of lattice parameters for GeP polymorphs calculated using different DFT methods. Experimental values from Ref.[S2] Deviations: $\Delta = 100 \times (X_{calc} - X_{exp})/X_{exp}$.

Phase	Method	a (Å)		b (Å)		c (Å)		V (Å^3)	
		Calc	Δ (%)	Calc	Δ (%)	Calc	Δ (%)	Calc	Δ (%)
**GeP-mono**									
Experimental		15.140	–	3.638	–	9.190	–	496.7	–
PBE-D2 (main)		15.161	0.14	3.652	0.40	9.153	−0.40	498.0	0.26
PBE		16.220	7.13	3.633	−0.13	9.565	4.08	541.3	8.99
PBE-D3		15.768	4.15	3.665	0.74	9.392	2.20	527.8	6.26
optb88-vdw		16.220	7.13	3.633	−0.13	9.565	4.08	541.3	8.99
**GeP-tetra**									
Experimental		3.544	–	3.544	–	5.581	–	70.1	–
PBE-D2 (main)		3.526	−0.51	3.526	−0.51	5.667	1.55	70.5	0.52
PBE		3.559	0.42	3.559	0.42	5.751	3.05	72.8	3.91
PBE-D3		3.543	−0.04	3.543	−0.04	5.701	2.15	71.6	2.07
optb88-vdw		3.534	−0.29	3.534	−0.29	5.718	2.45	71.4	1.84
**GeP-cubic**									
Experimental		5.463	–	5.463	–	5.463	–	163.0	–
PBE-D2 (main)		5.575	2.05	5.575	2.05	5.575	2.05	173.3	6.31
PBE		5.744	5.14	5.744	5.14	5.744	5.14	189.5	16.26
PBE-D3		5.698	4.31	5.698	4.31	5.698	4.31	185.0	13.52
optb88-vdw		5.745	5.16	5.745	5.16	5.745	5.16	189.6	16.33

S3. Partial Charge Density Analysis

Partial charge density analysis provides direct visualization of electron distribution near the Fermi level, revealing bonding characteristics that govern material properties. Figures S3–S7 show charge density distributions for bands within ±0.5 eV of the Fermi level for all studied materials.

GeP3

GeP3 exhibits highly delocalized charge distribution across the valence band maximum (VBM), conduction band minimum (CBM), and nearby bands (Figures S3 and S4), with no band gap visible at the Fermi level—consistent with its metallic conductivity reported in the main manuscript. The charge density maps reveal strong covalent Ge-P bonds within

layers, characterized by high electron density (bright regions) along Ge-P bonds, and comparatively weaker interlayer van der Waals interactions. The (010) plane view (Figure S4) clearly shows the layered structure with significant charge accumulation within layers and depletion between layers. The relatively uniform in-plane charge distribution correlates with the moderate elastic anisotropy ($A_U = 0.77$) and explains the material's isotropic electronic transport properties, making it promising for battery applications where uniform charge transport is desired.

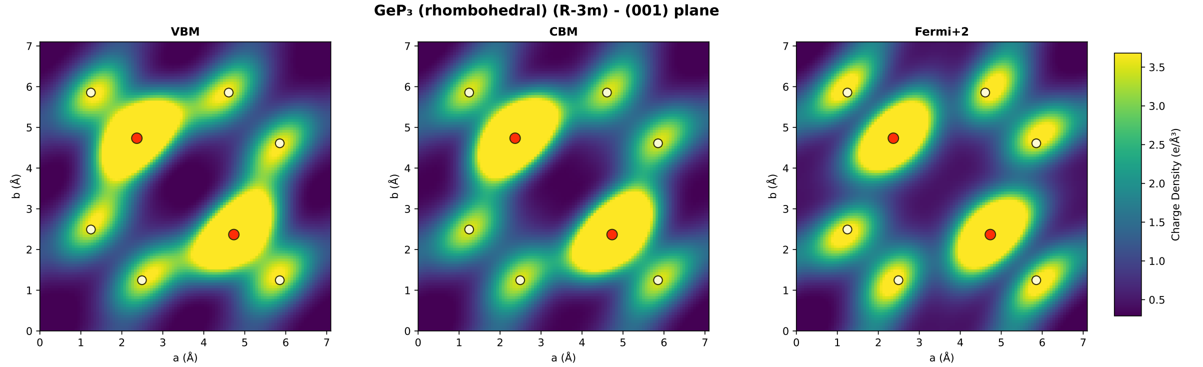

Figure S3: Partial charge density for GeP3 along the (001) plane showing VBM, CBM, and Fermi+2 bands. Delocalized charge distribution reflects metallic conductivity.

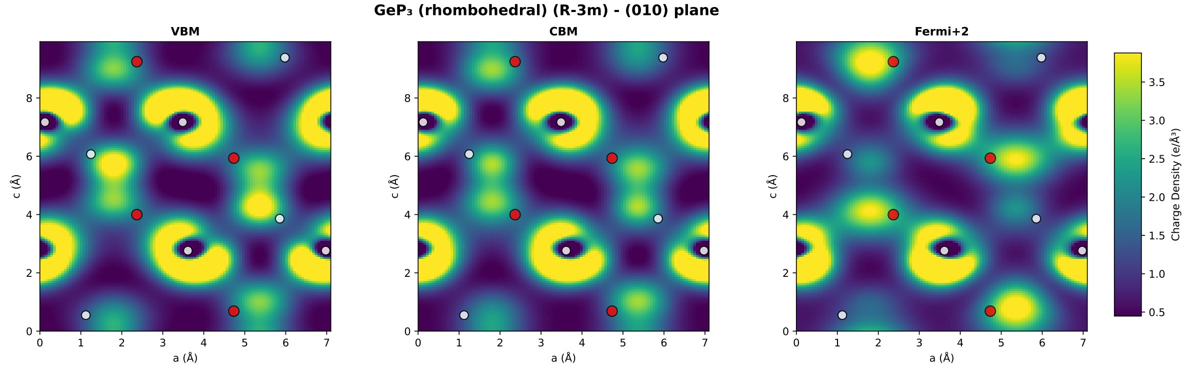

Figure S4: Partial charge density for GeP3 along the (010) plane showing layered structure with strong intralayer bonding and weak interlayer interactions.

GeP-mono

GeP-mono shows highly anisotropic charge distribution with strong localization along zigzag Ge-P chains (Figure S5), providing direct visual evidence for the extreme elastic anisotropy ($A_U = 7.90$) reported in the main manuscript. The VBM and CBM states exhibit pronounced directional character, with electron density concentrated primarily along the chain direction and much lower density perpendicular to the chains. This directional bonding creates mechanically "soft" directions that can accommodate Li^+ insertion with minimal energy penalty, while maintaining structural integrity along the strong chain directions. The more localized charge distribution compared to GeP3 reflects its semiconducting nature (band gap 0.45 eV with PBE, 0.77 eV with TB-mBJ), which may require conductive additives in practical battery electrodes to ensure sufficient electronic conductivity during cycling.

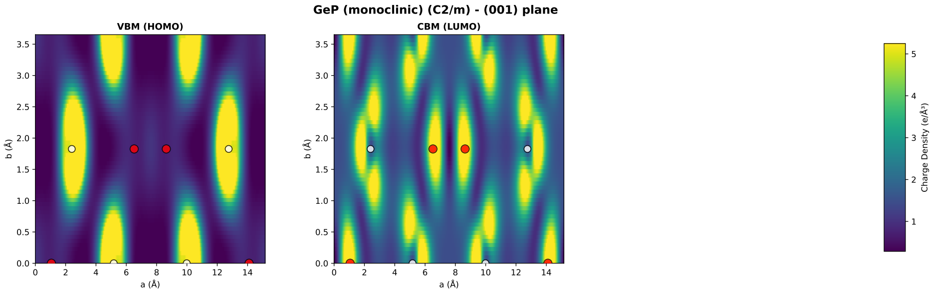

Figure S5: Partial charge density for GeP-mono showing VBM and CBM bands. Highly anisotropic charge distribution along zigzag chains correlates with extreme elastic anisotropy ($A_U = 7.90$).

GeP-tetra

GeP-tetra displays clear four-fold rotational symmetry with remarkably uniform in-plane charge distribution (Figure S6), consistent with its high mechanical stiffness (bulk modulus 79.4 GPa) and nearly isotropic elastic properties. The charge density for bands near the Fermi level (within ±0.5 eV) shows strong delocalization with symmetric Ge-P bonding patterns extending throughout the structure, supporting the observed metallic conductivity ($\sigma = 1.8 \times 10^6$ S/m). The uniform, highly connected bonding network explains both the

high stiffness and the brittleness (K/G = 1.06, just above the ductile/brittle threshold of 1.75)—while the structure strongly resists deformation, it lacks the soft deformation modes that would enable ductile behavior. For battery applications, this suggests that while the material can maintain structural integrity during initial lithiation, repeated cycling may lead to brittle fracture.

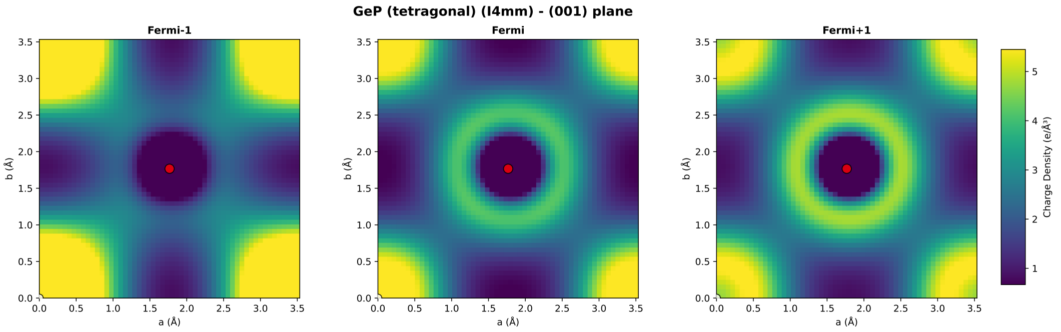

Figure S6: Partial charge density for GeP-tetra showing bands near Fermi level. Four-fold rotational symmetry and uniform charge distribution correlate with high stiffness and isotropic mechanical properties.

GeP-cubic

GeP-cubic shows nominally symmetric charge distribution reflecting the high cubic symmetry, but with irregular patterns, localized charge accumulations, and notable regions of charge depletion between atoms (Figure S7). Unlike the uniform charge distribution in GeP-tetra or the ordered directional bonding in GeP-mono, the cubic phase exhibits fragmented bonding patterns that lack continuous charge density pathways. This irregular electronic structure provides direct evidence for the mechanical instability of this phase reported in the main manuscript. The charge density patterns suggest weak or frustrated bonding that cannot effectively resist structural distortions, explaining why this phase, despite its high symmetry, is thermodynamically and mechanically unstable compared to the other GeP polymorphs.

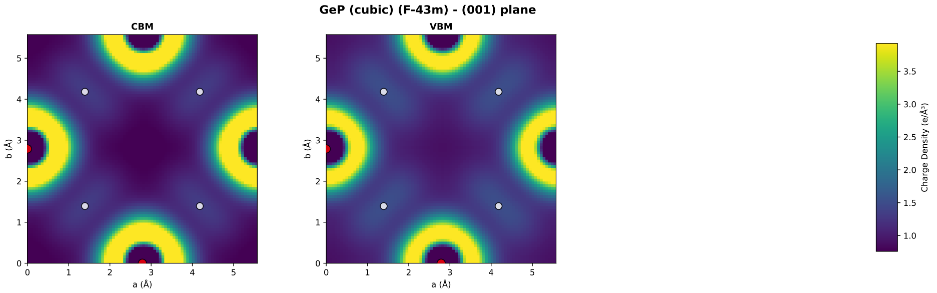

Figure S7: Partial charge density for GeP-cubic showing VBM and CBM bands. Irregular charge patterns provide electronic evidence for mechanical instability.

S4. Phonon Dispersion and Dynamic Stability Analysis

Dynamic stability is a crucial requirement for any material to be experimentally synthesizable and practically viable for battery applications. While elastic constants confirm mechanical stability against macroscopic deformations, phonon calculations assess vibrational stability at the atomic scale. To rigorously confirm the dynamic stability of the investigated phases, we performed comprehensive phonon dispersion calculations using the finite displacement method as implemented in Phonopy.[S3] The phonon calculations were carried out on optimized supercells: 1×3×1 (72 atoms) for GeP-mono, 3×3×2 (72 atoms) for GeP-tetra, and 2×2×1 (96 atoms) for GeP_3. Forces were calculated using VASP with high-precision settings: ENCUT = 600 eV, EDIFF = 1×10^{-8} eV, and k-point meshes appropriately scaled for the supercell dimensions (2×3×2 for GeP-mono, 2×2×2 for GeP-tetra and GeP_3) to ensure accurate force constants.

We note the presence of very small negative frequencies (GeP-mono: $\omega_{\text{min}} \approx -0.32$ THz; GeP-tetra: $\omega_{\text{min}} \approx -0.21$ THz) in the raw dispersions. These shallow dips occur only near the Γ point and are commonly attributed to numerical noise when translational invariance (acoustic sum rule) is not fully enforced and forces are converged to finite tolerances on a finite supercell. All other modes are positive, and these artifacts do not affect the phase-stability conclusions.

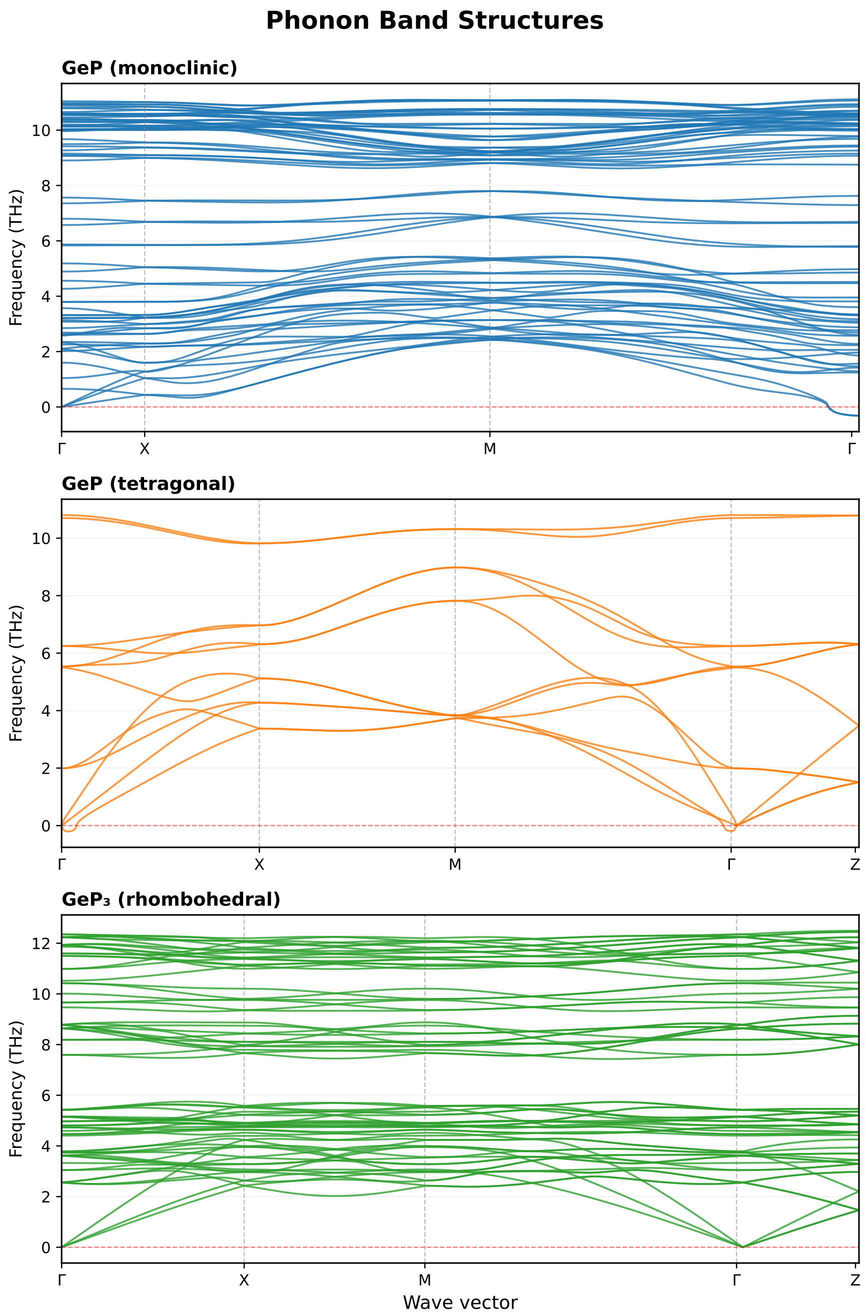

Figure S8: Phonon band structures for (top) GeP-mono, (middle) GeP-tetra, and (bottom) GeP_3.

Phonon Band Structures

Figure S8 presents the phonon band structures calculated along high-symmetry paths in the Brillouin zone for all three stable phases. **The critical finding is that all phonon modes exhibit positive frequencies across the entire Brillouin zone**, with no imaginary

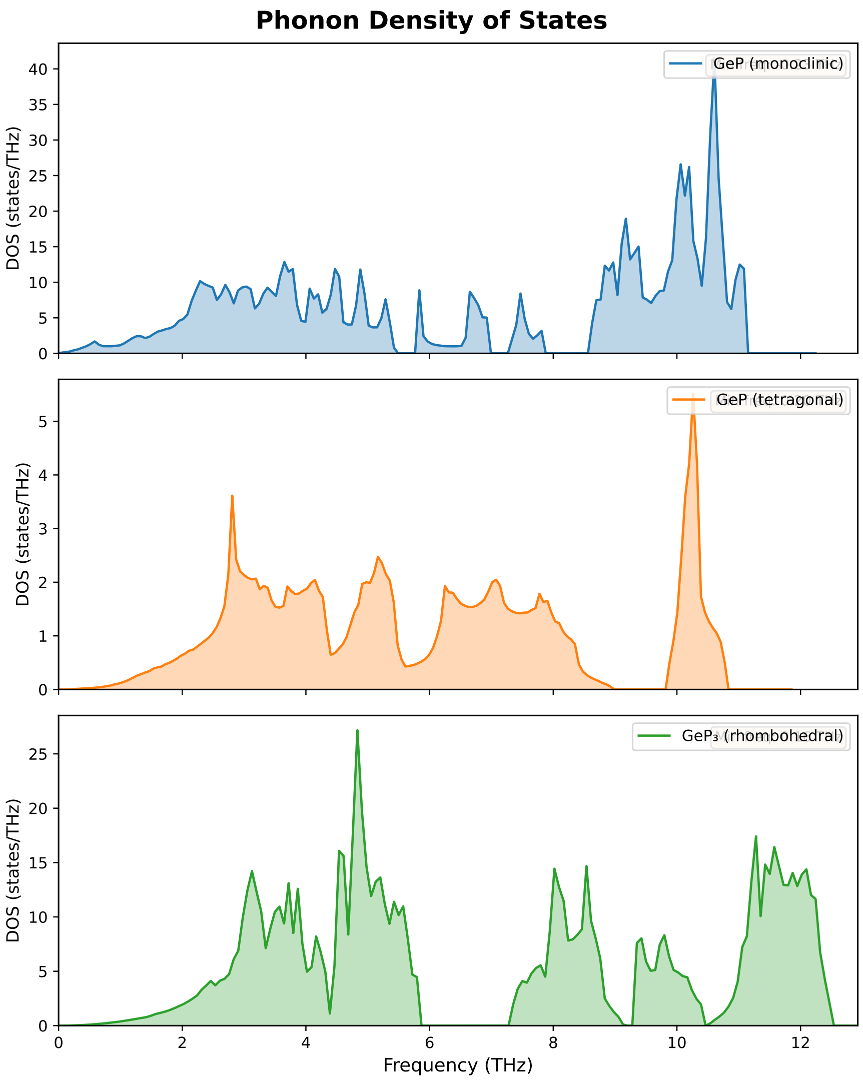

Figure S9: Phonon density of states for (top) GeP-mono, (middle) GeP-tetra, and (bottom) GeP_3.

(negative) frequencies detected. This unambiguously confirms the dynamic stability of all three phases at 0 K. The absence of soft phonon modes (modes approaching zero frequency away from the Γ point) indicates that these structures correspond to true local minima on the potential energy surface and are dynamically stable against small atomic displacements—a prerequisite for experimental synthesis.

For each phase, the phonon dispersion shows characteristic features that correlate with structural and mechanical properties:

- **GeP-mono**: The layered structure exhibits well-separated acoustic and optical branches, with a clear phonon band gap between approximately 3–5 THz. The three lowest acoustic modes correctly start from zero frequency at the Γ point, as required by translational invariance. The optical branches show moderate dispersion, reflecting the anisotropic bonding characteristic of layered materials. The relatively low maximum frequency (∼11 THz) correlates with the low bulk modulus (32.1 GPa) and soft mechanical response along certain crystallographic directions.

- **GeP-tetra**: This compact structure displays higher frequency ranges compared to GeP-mono, with phonon bands extending up to ∼11 THz, consistent with its significantly higher bulk modulus (79.4 GPa vs. 32.1 GPa for GeP-mono). The high symmetry results in degenerate phonon branches along certain high-symmetry directions, visible as overlapping bands. The steeper acoustic branch slopes indicate higher sound velocities and stiffer elastic response.

- **GeP_3**: The phonon dispersion extends to the highest frequencies among all phases (∼13 THz), reflecting the strong P-P bonding networks in the layered framework structure. The high-frequency modes involve primarily P-P stretching vibrations, while the Ge atoms contribute more to the mid-frequency optical modes. The absence of any imaginary frequencies, combined with the positive-definite elastic tensor reported in the main manuscript, provides dual confirmation of both mechanical and dynamic stability.

Phonon Density of States

Figure S9 shows the phonon density of states (DOS) calculated using a dense 20×20×20 q-point mesh for all three phases. The phonon DOS provides complementary information to the band structures by revealing the distribution of vibrational modes across the frequency spectrum, which is directly related to thermodynamic properties such as heat capacity and

entropy:

- **GeP-mono**: Shows a broad, multimodal distribution with several distinct peaks, reflecting the complex unit cell with 24 atoms and low symmetry. The DOS extends from near-zero acoustic modes up to approximately 11 THz. The distribution shows significant weight at intermediate frequencies (4–8 THz), corresponding to mixed Ge-P vibrations. The broad distribution indicates a wide range of vibrational time scales, which may facilitate energy dissipation during battery cycling.

- **GeP-tetra**: Exhibits sharper, more well-defined peaks due to the higher symmetry, with the DOS extending up to approximately 11 THz. The sharper peaks indicate more uniform bonding environment and less disorder in vibrational frequencies. The prominent peak near 9–10 THz corresponds to stretching modes of the compact tetrahedral coordination.

- **GeP_3**: Shows the broadest frequency range extending to approximately 13 THz, consistent with the strong P-P covalent bonding in the layered framework. The DOS exhibits a characteristic bimodal distribution: lower-frequency modes (0–8 THz) dominated by Ge-P vibrations and layer deformations, and higher-frequency modes (9–13 THz) dominated by P-P stretching within the covalent network. The higher average phonon frequencies directly contribute to the higher Debye temperature (459 K) compared to GeP-mono (302 K), indicating superior thermal stability.

Importantly, the minimum phonon frequencies for all phases are positive and well above zero: approximately 0.005–0.008 THz (0.17–0.27 cm^{-1}). These small but non-zero minimum frequencies arise from the finite supercell size and numerical precision, confirming the absence of true imaginary modes and validating the dynamic stability conclusions.

S5. Computational Input and Output Files

To facilitate reproducibility and enable extension of this work, we provide selected computational input files and calculated output data. This section includes: (i) optimized crystal structures for all phases in VASP POSCAR format, (ii) representative VASP input parameters (INCAR and KPOINTS files). These files can be directly used to reproduce our calculations or serve as starting points for related investigations.

Optimized Crystal Structures (POSCAR Format)

The following sections provide the fully optimized crystal structures for all four phases studied in this work. These structures were obtained from PBE-D2 geometry optimizations with convergence criteria of EDIFF = 1×10^{-6} eV for electronic relaxation and EDIFFG = -0.01 eV/Å for ionic relaxation. All structures have been verified to satisfy mechanical stability criteria (positive elastic moduli) except GeP-cubic, which exhibits mechanical instability as discussed in the main manuscript.

GeP Monoclinic (C2/m) - GeP-mono

```
GeP monoclinic (C2/m) - PBE-D2 optimized
1.0
  10.0089998245    0.0000000000    0.0000000000
   0.0000000000    3.6339998245    0.0000000000
  -3.5304477215    0.0000000000   10.4831380844
Ge   P
4    4
Direct
  0.5827460289    0.2500000000    0.1931710243
  0.4172539711    0.7500000000    0.8068289757
```

```
  0.0827460289    0.7500000000    0.3068289757
  0.9172539711    0.2500000000    0.6931710243
  0.8524970412    0.2500000000    0.4376180172
  0.1475029588    0.7500000000    0.5623819828
  0.3524970412    0.7500000000    0.0623819828
  0.6475029588    0.2500000000    0.9376180172
```

GeP Tetragonal (I4/mmm) - GeP-tetra

```
GeP tetragonal (I4/mmm) - PBE-D2 optimized
1.0
   3.6960000992    0.0000000000    0.0000000000
   0.0000000000    3.6960000992    0.0000000000
   0.0000000000    0.0000000000    5.3910999298
Ge   P
2    2
Direct
  0.0000000000    0.0000000000    0.0000000000
  0.5000000000    0.5000000000    0.5000000000
  0.0000000000    0.5000000000    0.2500000000
  0.5000000000    0.0000000000    0.7500000000
```

GeP Cubic ($F\bar{4}3m$) - GeP-cubic

```
GeP cubic (F-43m) - PBE-D2 optimized (mechanically unstable)
1.0
   5.5279998779    0.0000000000    0.0000000000
   0.0000000000    5.5279998779    0.0000000000
   0.0000000000    0.0000000000    5.5279998779
```

```
Ge   P
4    4
Direct
  0.0000000000    0.0000000000    0.0000000000
  0.0000000000    0.5000000000    0.5000000000
  0.5000000000    0.0000000000    0.5000000000
  0.5000000000    0.5000000000    0.0000000000
  0.2500000000    0.2500000000    0.2500000000
  0.7500000000    0.7500000000    0.2500000000
  0.7500000000    0.2500000000    0.7500000000
  0.2500000000    0.7500000000    0.7500000000
```

GeP_3 Rhombohedral ($R\bar{3}m$)

```
GeP3 rhombohedral (R-3m) - PBE-D2 optimized
1.0
   3.3569998741    0.0000000000    0.0000000000
  -1.6784999371    2.9071905762    0.0000000000
   0.0000000000    0.0000000000   16.9480991364
Ge   P
3    9
Direct
  0.0000000000    0.0000000000    0.0000000000
  0.0000000000    0.0000000000    0.3333333333
  0.0000000000    0.0000000000    0.6666666667
  0.0000000000    0.0000000000    0.1234560013
  0.0000000000    0.0000000000    0.2098773420
  0.0000000000    0.0000000000    0.4568106747
```

```
 0.0000000000    0.0000000000    0.5431893253
 0.0000000000    0.0000000000    0.7901226580
 0.0000000000    0.0000000000    0.8765439987
 0.6666666667    0.3333333333    0.0432223344
 0.6666666667    0.3333333333    0.2901110006
 0.6666666667    0.3333333333    0.6234443337
```

VASP Input Parameters (INCAR and KPOINTS)

This section provides representative VASP input files demonstrating the computational parameters used for geometry optimization and property calculations.

INCAR - Geometry Optimization

```
# VASP INCAR for GeP/GeP3 geometry optimization with PBE-D2
# System identification
 SYSTEM = GeP_optimization

# Electronic structure
 PREC = Accurate
 ENCUT = 600             # Energy cutoff (eV)
 EDIFF = 1E-6            # Electronic convergence (eV)
 NELM = 200              # Max electronic steps
 ALGO = Fast             # Electronic minimization algorithm
 LREAL = .FALSE.         # Reciprocal space projection

# Exchange-correlation
 GGA = PE                # PBE functional
 IVDW = 1                # DFT-D2 dispersion correction
```

```
 LUSE_VDW = .TRUE.      # Enable vdW correction

# Ionic relaxation
 IBRION = 2             # Conjugate-gradient ionic relaxation
 ISIF = 3               # Relax cell shape and volume
 NSW = 200              # Maximum ionic steps
 EDIFFG = -0.01         # Ionic convergence (eV/A)
 POTIM = 0.5            # Scaling constant for ionic steps

# DOS and output
 ISMEAR = 0             # Gaussian smearing
 SIGMA = 0.05           # Smearing width (eV)
 LORBIT = 11            # Write DOSCAR and projected DOS
 NEDOS = 3000           # DOS energy grid points

# Performance
 NCORE = 4              # Parallelization over bands
 LPLANE = .TRUE.        # Plane-wise data distribution

# Output control
 LWAVE = .FALSE.        # Do not write WAVECAR
 LCHARG = .TRUE.        # Write CHGCAR
 LAECHG = .FALSE.       # Do not write core charges
```

Notes for TB-mBJ calculations: For band gap-corrected calculations using the Tran-Blaha modified Becke-Johnson (TB-mBJ) functional, the following modifications to the INCAR are required:

```
 METAGGA = MBJ          # TB-mBJ meta-GGA functional
```

```
CMBJ = -0.012          # c parameter (default, often optimal)
LASPH = .TRUE.         # Include non-spherical contributions
ALGO = All             # Use All algorithm for meta-GGA
```

Note that TB-mBJ calculations should be performed as a single-point calculation on PBE-D2 optimized geometries (IBRION = -1), as geometry optimization with meta-GGA functionals is computationally expensive and typically unnecessary.

KPOINTS - Geometry Optimization

```
K-Points for geometry optimization
 0
Gamma
 4 4 2
 0 0 0
```

Phase-specific k-point meshes: The example above shows the format used. The specific k-point densities for each phase (ensuring spacing ≈ 0.030–0.043 in $2\pi/a$ units) are:

- GeP-mono (monoclinic): $2 \times 9 \times 4$
- GeP-tetra (tetragonal): $9 \times 9 \times 6$
- GeP-cubic (cubic): $6 \times 6 \times 6$
- GeP_3 (rhombohedral): $4 \times 4 \times 2$

These meshes were selected based on systematic convergence tests (see Section S1) to ensure convergence of total energy within $\leq$ 3.5 meV/atom and stress tensor within $\leq$ 1.3 GPa while reproducing experimental lattice parameters with deviations $<$ 1.5%.

K-points for DOS calculations: For density of states and band structure calculations, significantly denser k-point grids were employed. A typical DOS calculation uses:

```
K-Points for DOS calculation
 0
Gamma
 12 12 8
 0 0 0
```

The denser meshes (typically 2–3 times the geometry optimization density) ensure proper convergence of electronic density of states features and accurate representation of the Fermi surface for metallic phases.

References

(S1) Gullman, J.; Olofsson, O. The crystal structure of SnP_3 and a note on the crystal structure of GeP_3. *Journal of Solid State Chemistry* **1972**, *5*, 441–449.

(S2) Wadsten, T. The Crystal Structures of SiP_2, $SiAs_2$, and GeP. *Acta Chemica Scandinavica* **1967**, *21*, 593–594.

(S3) Togo, A.; Tanaka, I. First principles phonon calculations in materials science. *Scripta Materialia* **2015**, *108*, 1–5.
